# Systematic computation with functional gene-sets among leukemic and hematopoietic stem cells reveals a favorable prognostic signature for acute myeloid leukemia

**DOI:** 10.1186/s12859-015-0510-7

**Published:** 2015-03-24

**Authors:** Xinan Holly Yang, Meiyi Li, Bin Wang, Wanqi Zhu, Aurelie Desgardin, Kenan Onel, Jill de Jong, Jianjun Chen, Luonan Chen, John M Cunningham

**Affiliations:** 1 Department of Pediatrics, and Comer Children’s Hospital, Section of Hematology/Oncology, The University of Chicago, 900 East 57th Street, KCBD Room 5121, Chicago, Illinois 60637 USA; 2 Key Laboratory of Systems Biology, Institute of Biochemistry and Cell Biology, Shanghai Institutes for Biological Sciences, Chinese Academy of Sciences, Shanghai, China; 3 Department of Medicine, The University of Chicago, Chicago, USA; 4 Laboratory Schools, The University of Chicago, Chicago, USA

**Keywords:** Functional gene-set, Dynamic network biomarker, Relative effect analysis, Leukemic stem cell, AML outcome

## Abstract

**Background:**

Genes that regulate stem cell function are suspected to exert adverse effects on prognosis in malignancy. However, diverse cancer stem cell signatures are difficult for physicians to interpret and apply clinically. To connect the transcriptome and stem cell biology, with potential clinical applications, we propose a novel computational “gene-to-function, snapshot-to-dynamics, and biology-to-clinic” framework to uncover core functional gene-sets signatures. This framework incorporates three function-centric gene-set analysis strategies: a meta-analysis of both microarray and RNA-seq data, novel dynamic network mechanism (DNM) identification, and a personalized prognostic indicator analysis. This work uses complex disease acute myeloid leukemia (AML) as a research platform.

**Results:**

We introduced an adjustable “soft threshold” to a functional gene-set algorithm and found that two different analysis methods identified distinct gene-set signatures from the same samples. We identified a 30-gene cluster that characterizes leukemic stem cell (LSC)-depleted cells and a 25-gene cluster that characterizes LSC-enriched cells in parallel; both mark favorable-prognosis in AML. Genes within each signature significantly share common biological processes and/or molecular functions (empirical p = 6e-5 and 0.03 respectively). The 25-gene signature reflects the abnormal development of stem cells in AML, such as *AURKA* over-expression. We subsequently determined that the clinical relevance of both signatures is independent of known clinical risk classifications in 214 patients with cytogenetically normal AML. We successfully validated the prognosis of both signatures in two independent cohorts of 91 and 242 patients respectively (log-rank p < 0.0015 and 0.05; empirical p < 0.015 and 0.08).

**Conclusion:**

The proposed algorithms and computational framework will harness systems biology research because they efficiently translate gene-sets (rather than single genes) into biological discoveries about AML and other complex diseases.

**Electronic supplementary material:**

The online version of this article (doi:10.1186/s12859-015-0510-7) contains supplementary material, which is available to authorized users.

## Background

Acute myeloid leukemia (**AML**) has a high mortality rate. Leukemic stem cells (**LSC**) represent a rare self-renewing cellular subpopulation in each AML. Their chemo-resistant properties are associated with adverse outcomes [1,2]. However, the key events that confer stem cell-like characteristics to LSCs remain unclear. Still, gene expression markers for LSC among studies lack reproducibility, although the characterization of these genes is likely to reveal novel and tractable targets to improve treatment outcomes [3-5].

We hypothesize that one main limitation is that there is no definitive method to isolate LSC from bulk cell samples, and therefore, methods attempting to identify LSC signature are limited by cell heterogeneity. To overcome this problem, we have developed a novel computational systems biology analysis framework. This framework has a four-fold advantage: i) It enables multi-resource data integration and biologically functional interpretation by working on the scale of functional gene-sets; ii) It defines a cluster of functionally interpretable gene-sets shared among LSC populations generated by different labs; iii) It identifies mutual functional relationships from a network of spatiotemporally (disease developmentally) dynamic expression; iv) It provides a personalized leukemic prognostic indicator derived from the identified gene-set clusters. Here, we present the method and demonstrate its application to identify a common LSC signature. The identified LSC-associated biomarkers have a direct link to biological interpretation and clinical application.

First, to facilitate multi-resource data integration and biologically functional interpretation, knowledge-centric analysis (or the analysis of gene-sets) was developed by us and others, e.g., GSEA (Gene Set Enrichment Analysis) [6-8]. The analysis of gene-sets is superior to single-gene analysis in regard to noise and dimension reduction, as well as its desired biological interpretability [9]. However, most gene-set analysis methods impose inherent limits on low cross-dataset comparison or reproducibility from gene-by-sample measurements, as only the genes measured by all collected platforms can be interrogated together. Improved gene-set analyses condense transcriptomic data from gene-by-sample measurements (gene profiles) to gene-set-by-sample measurements (gene-set profiles), which are gene-coverage-difference tolerable and a breakthrough in genome analytics coordinates. Such gene-set-by-sample analyses facilitate the integration and analysis of multiple datasets, platforms, or layers of omics-data, by assigning them into a uniform gene-set scale [10-13]. Some transcription-focused methods, such as **GSVA** (Gene Set Variation Analysis), condense gene expression values into gene-set scores by evaluating sample-wise enrichments. Yet an open question is the sample-wised statistics before calculating gene-set scores, which limits a mechanistic representation of individual samples. To overcome these deficiencies, we have developed a gene-set-by-sample algorithm, **FAIME** (Functional Analysis of Individual Microarray (or RNA-seq) Expression) [7,8]. Note that for each sample, FAIME compares the cumulative effects of genes inside a gene-set with the effects of those outside. Additionally, **FAIME** employs an expression-based weight to rectify biases introduced by low-valued genes [14], and thus quantifies gene-sets primarily according to its highly expressed gene members. However, sensitivity remains a challenge as, at a significance level of false discovery rate (FDR) of 0.05, FAIME could identify hundreds of gene-sets, an impractical number for wet-lab validation. Therefore, we introduce in this study a new weighting parameter into the FAIME algorithm to better control the type-I error, especially for small gene-sets. Additionally, the integration of microarray and RNA-seq data is a new task that we have performed in this study, given the increase in their publication and availability.

Second, dynamic network biomarker (DNB) analysis has been developed on the gene level to address the challenge of temporal and spatial gene expression profiling. It identifies the disease biomarkers leading the whole system from the normal state to a disease state [15,16]. Here, we demonstrate the first dynamic network biomarker analysis on the gene-set level (termed dynamic network mechanism – **DNM** analysis). These gene-set-based network-relationship dynamics (rather than the static featured single gene-set up- or down-regulations) translate seemingly uninterpretable genomic data into distinct clinical prognoses. In particular, the identified gene-sets define a new biomarker for the characterized stem cell sub-population.

Finally, the goal of cancer treatment is to improve outcomes by earlier diagnosis and targeted therapy for each patient. We have developed the relative expression concept to build a personalized prognostic indicator on the gene level [17,18]. In this study, we expand the concept of relative expression to gene-set clusters and identify a prognostic indicator evaluated by three large cohorts. Meta-analysis is a powerful solution in identifying common LSC signatures shared among different LSC subpopulations with the challenge of small sample size and high cell heterogeneity. In this study, we pool sorted samples from eight labs, including microarray and RNA-seq data, into three groups: **HSC+** (verified hematopoietic stem cells enriched, n = 23), **LSC+** (LSC enriched, n = 77), and **LSC-** (LSC depleted, n = 59) cells. Enlarged sample size ensures reliable detection.

**Figure 1:**
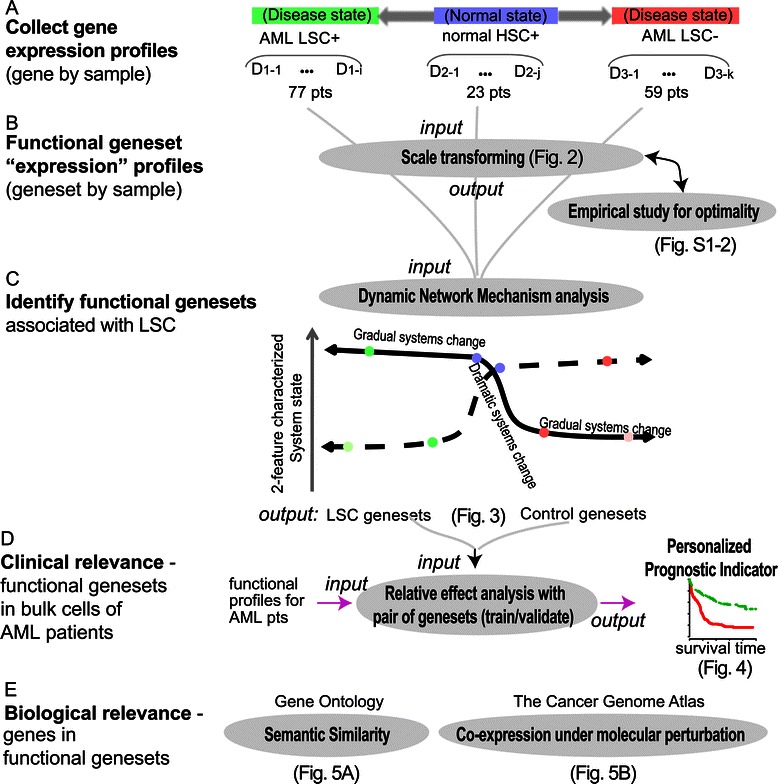
**Flowchart of normalization and analysis of pooled molecular function profiles.** Panel **A)** Collection of gene expression profiles. Datasets (D) of Acute Myeloid Leukemia (AML) positive or negative (1 and 2 respectively) for Leukemia Stem Cell (LSC), and of normal Hematopoietic Stem Cells (HSC) (3). Panel **B)** Calculation of gene-set profiles. Panel **C)** Identification of functional gene-sets associated with LSCs using the dynamic network mechanism analysis. A cartoon shows that different features characterize the dramatic systems stage changes in different ways. Panel **D)** Evaluation of clinical relevance in primary AML samples. Panel **E)** Evaluation of biological relevance using independent data resources.

Molecular Signature Database (**MSigDB**) [19] is the most popular gene-set database that defines groups of genes associated with a common function, pathway or other characteristics using biological evidence. Built on MSigDB-defined gene-sets and the above four strategies (Figure 1), we generated gene-set profiles using FAIME (with a weighting parameter) and identified a cluster of three gene-sets with 30 genes representative for LSC-. We also generated gene-set profiles using the GSVA method and identified another cluster of three functional gene-sets with 25 genes in LSC+. The genes within each cluster of gene-sets display simultaneous co-variation with high mutual correlation exclusively within the corresponding cell subpopulations. Using available samples of primary AML patients, we further assessed the clinical relevance by prognosis and the biological relevance by ontology for both gene sets.

## Results and discussion

### Improved functional gene set profiles and the inter-dataset normalization allow cross-dataset comparisons

**Figure 2:**
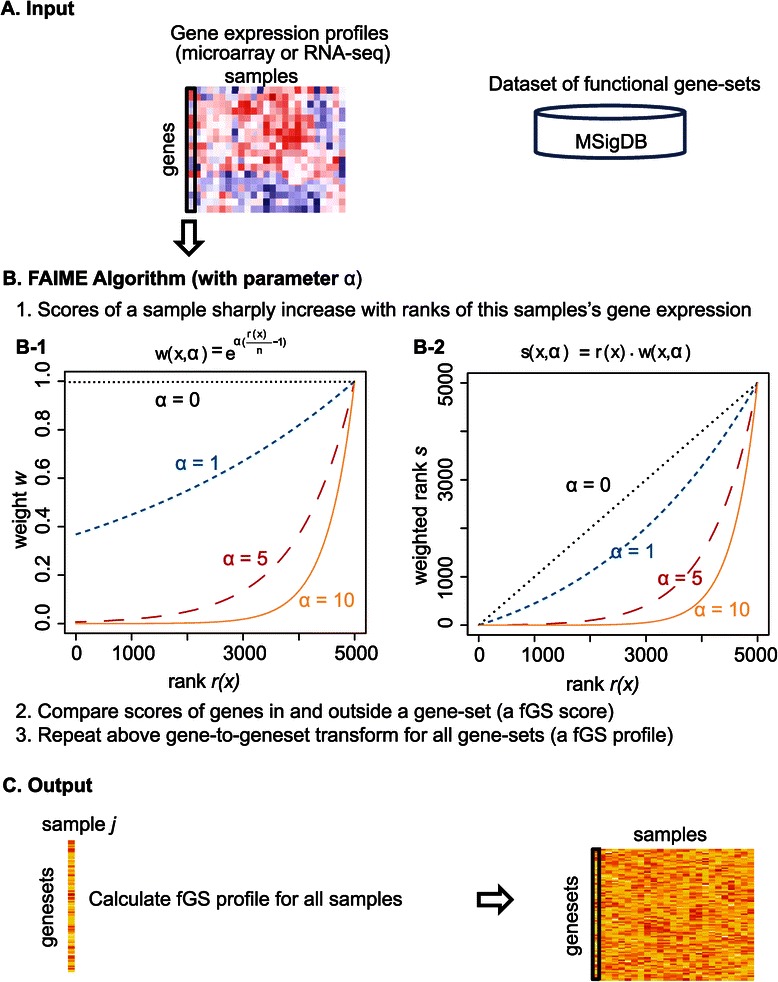
**FAIME algorithm with a new parameter α outline.** Panel **A)** The input for the FAIME algorithm is either a gene expression matrix in the form of log2 microarray expression values or RNA-seq counts, and a database of gene-sets. Panel **B)** Mechanism (or gene-set) score is defined as the difference between the scored expression of genes inside and outside a previously defined gene-set. B-1) Applying an increasingly larger α to the FAIME method. The weight (y-axis) is an exponential function of gene expression ranks (x-axis) adjusted by the parameter α. B-2) Weight-dependent qualitative scores sharply increase with gene rank. The score (y-axis) is the product of gene expression ranks (x-axis) and the rank’s weight adjusted by the parameter α. In each panel, the more highly expressed genes are ranked higher on the x-axis. The dashed line represents the score obtained with no weighting (*i.e.*, ranking only). Panel **C)** Output of the algorithm as a matrix containing mechanism scores for each gene-set and sample.

Some GSEA algorithms (e.g., the Bioconductor package PGSEA) suggest an applicable gene-set (GS) size ranging from 25 to 500 genes. However, half of the canonical pathways have only 10–30 gene members (MSigDB). To control the type-I error when testing on small GSs effectively, we improved the FAIME algorithm with a new adjusted parameter α (hereafter termed as FAIME.5 when α = 5). The larger α is, the higher the weight for the genes at the extreme top of expression ranks is, exponentially (Figure 2B). The parameter α thus acts as a “soft threshold” [20]. Previous studies have shown that both GSVA and FAIME exhibit better or comparable statistical power to that of the GSEA algorithm [7,21]. We compared FAIME.α with GSEA and GSVA in a simulation study. By empirically testing three integers (1,5, and 10) as candidate α values, we chose α = 5. FAIME.5 provided a better control of type-I errors than FAIME.1 but a higher accuracy than FAIME.10 for small GSs with less than 30 genes (Additional file 1: Figure S1, Additional file 2: Text S1: S Results 1.1). Even GSEA, FAIME and GSVA identified distinct GSs in an initial two-group comparison test, FAIME.1 and FAIME.5 shared over half of their identified GSs, and the FAIME shared more identified GSs with GSEA than GSVA (data not shown). Given that FAIME and GSVA have exhibited better or comparable statistical power to that of the GSEA algorithm in certain conditions of previous studies [7,21], we used FAIME.5 and GSVA in the subsequent LSC study. A vignette file with R source codes (Additional file 3: Text S2) shows how to run FAIME.α on a Bioconductor available gene expression dataset, using the MSigDB defined gene-sets of CGP (chemical and genetic perturbations) as a demo.

The required sample size for functional gene set analysis was also discussed in the simulation study. With at least 20 samples per group, the three methods (FAIME, GSEA, and GSVA) could identify repeatable signatures in the scenarios in which half or more of genes in a GS exhibit strong signal-to-noise ratio (Additional file 1: Figure S1- C,D, dashed lines). Therefore, to ensure statistical power, we collected more than 20 samples per group (of microarray or RNA-seq data (Table 1 and Additional file 4: Table S1) - and performed meta-analysis on the GS level. Meta-analysis is one solution for the limited number of LSC+ sub-populations and their heterogeneity across studies. This study further meets an outstanding need to characterize LSC because, on average, only 16% (2%-28%, Additional file 1: Figure S2A) of reported LSC+ gene signatures were repeatable across five previous studies that attempted to distinguish malignant LSC in AML [1,22-24].

**Table 1:** **Summary of collected transcriptional and clinical data for AML LSC+, AML LSC-, and normal HSC+ samples**

	**GAL**	**CBX21**	**GSE24006**	**GSE30377**	**GSE17054**	**ETABM978**	**GSM651554**	**Group**
1st author	**Gal**	**Ishikawa F**	**Gentles AJ**	**Eppert K**	**Majeti R**	**Goardon N**	**Hu**	
Journal	Leukemia	JAMA	JAMA leukemia	Nature Med	PNAS	Cancer Cell	Genome Res	
Year	2006	2010	2011	2011	2009	2011	2011	
PMID	17039238	17952057	21177505	21873988	19218430	21251617	21795385	
**Engraftment verification for LSC+**	N/A	(NOD/SCID/IL)2r gamma (null) (NSG)	N/A	NOD/ShiLtSz-SCID (NODSCID)	N/A	NOD/SCID or NSG	N/A	
AML LCS+ (CD34 + CD38-		8		13				LSC+ (n = 77)
AML LCS+ (CD34-CD38-)				3				
AML LCS+ (CD34-CD38+)				1				
AML LCS+ (CD34 + CD38+)				8				
AML LCS+ (Lin-CD34 + CD38-CD90-)								
AML LCS+ (Lin-CD34 + CD38-CD90-CD45RA+)						22		
GMP-like AML LSC+ (Lin-CD34 + CD38 + CD123+/loCD110-CD45RA + CD45RA+)						22		
AML leukemia progenitor cell + (LPC+) (hCD34 + hCD38+)	5	8		5				LSC- (n = 59)
AML CMP+ (Lin-CD34 + CD38+)			7	1		1		
AML MPP+ (Lin-CD34 + CD38-CD90-CD45RA-)						2		
AML Blasts (Lin-CD34-)			7	23				
normal HSC+ (CD34 + CD133+)							1	normal HSC+ (n = 23)
normal HSC+ (Lin-CD34 + CD38-)								
normal HSC+ (Lin-CD34 + CD38-)								
normal HSC+ (Lin-CD34 + CD38loCD36-)								
normal HSC+ (Lin-CD34 + CD38-CD90+)					4			
normal HSC+ (Lin-CD34 + CD38-CD90 + CD45RA-)			7			5		

To make the samples per group from disparate resources comparable, we applied a straightforward inter-dataset normalization (z-transfer) on mechanism profiles that varied across observed datasets (Additional file 1: Figure S3). For both FAIME and GSVA methods, this inter-dataset normalization reduces the cross-sample variance regardless of gene-set sizes and re-scales gene-set scores to be symmetric and zero-centered (Additional file 1: Figure S3B). Subsequently, the majority of such normalized gene-set scores met the null hypothesis of conventional statistical models designed for gene expression, which is that few genes differ between phenotypes. Therefore, we can employ statistical models designed for gene expression profiles on these gene-set profiles, ensuring direct evaluation of gene-set significance with phenotypes. However, using the conventional hypergeometric test on 1320 canonical pathways each with five or more genes from the MSigDB (v4.0), we observed a large number exhibiting significant scores between two sub-populations (e.g., 210 FAIME-derived pathways meet the criteria of FDR < 0.05 and FC > 2, Bioconductor Limma package), suggesting the need for a proper model to concentrate on core pathways.

### Dynamic network mechanism (DNM) analysis defines new LSC-representative signatures

To explore critical disease developmental signatures using the gene-set profiles, we developed a dynamic network mechanism (**DNM**) analysis. The hypothesis is that the deteriorations of patient’s condition in complex diseases such as AML are abrupt during the progression of disease and may be caused by a critical transition at a tipping point [15]. DNM is built on a method, called dynamical network biomarker (DNB), previously designed to capture early-warning signals before a critical transition from normal state to disease state [15,16]. The DNM analysis identified gene-sets are hereafter referred to as **DNM gene-sets**. Given gene-set profiles of populations with different disease states, the DNM analysis searches for the critical sub-network that exhibits a low variability and high dependence intra-population, but high variability and low dependence inter-population. Specifically, we evaluated the network nodes (the gene-sets) by the standard deviations for variability and pairwise Pearson correlation coefficients for dependence among three different cell sub-populations: HSC+, LSC+, and LSC- cells. We thereafter identified the critical transitional sub-population and, subsequently, the best cluster of gene-sets to distinguish this sub-population from the other two using a combined score of the variability and dependence of gene-sets. As a result, a new 30-gene signature best distinguishes the AML LSC- sub-population based on FAIME.5 profiles, and a new 25-gene signature best distinguishes the AML LSC+ population based on GSVA profiles (Table 2). Note that the DNM identified critical transitional sub-population from FAIME gene-set profiles or GSVA gene-set profiles is different, suggesting cell diversity. The number of involved genes from identified gene-sets is a practical number for wet-lab validation. Each signature is a cluster of 3 or 4 gene-sets showing simultaneous co-variation with mutual correlation exclusively within the corresponding cell subpopulations.

**Table 2:** **Identified 30-gene and 25-gene signatures**

**Symbol**	**Entrez gene name**	**Type(s)^**	**Biomarker#^**	**Symbol**	**Entrez gene name**	**Type(s)^**	**Biomarker#^**
LSC- 30 genes				LSC+ 25 genes			
ANLN	anillin, actin binding protein	other		APPBP2	amyloid beta precursor protein (cytoplasmic tail) binding protein 2	other	
AURKA	aurora kinase A	kinase	E	ATXN3	ataxin 3	peptidase	
CCNA1	cyclin A1	other		CCND2	cyclin D2	other	D, E
CCL5	chemokine (C-C motif) ligand 5	cytokine	D, E, U	DYNLL2	dynein, light chain, LC8-type 2	other	
CD38	CD38 molecule	enzyme	E, P, U	ERC1	ELKS/RAB6-interacting/CAST family member 1	other	
CDC25B	cell division cycle 25B	phosphatase		ETV6	ets variant 6	transcription regulator	
CDK1	cyclin-dependent kinase 1	kinase		GGNBP2	gametogenetin binding protein 2	other	
CENPA	centromere protein A	other		GIMAP6	GTPase, IMAP family member 6	other	
CLC	Charcot-Leyden crystal galectin	enzyme		KIF1B	kinesin family member 1B	transporter	
CPA3	carboxypeptidase A3 (mast cell)	peptidase		KIF1C	kinesin family member 1C	other	
CSTA	cystatin A (stefin A)	other		KRAS	Kirsten rat sarcoma viral oncogene homolog	enzyme	D, E, P, R, U
DDX53	DEAD (Asp-Glu-Ala-Asp) box polypeptide 53	other		LOC728392	uncharacterized LOC728392	other	
DLGAP5	discs, large (Drosophila) homologassociated protein 5	phosphatase		MAF	v-maf avian musculoaponeurotic fibrosarcoma oncogene homolog	transcription regulator	
HGF	hepatocyte growth factor (hepapoietin A; scatter factor)	growth factor	D, DP, E, P, U	MPO	myeloperoxidase	enzyme	D, E, U
IL36B	interleukin 36, beta	cytokine		MTERFD2	MTERF domain containing 2	other	
KIAA0101	KIAA0101	other		NAV1	neuron navigator 1	enzyme	
LATS2	large tumor suppressor kinase 2	kinase		PIAS1	protein inhibitor of activated STAT, 1	transcription regulator	U
MBD3	methyl-CpG binding domain protein 3	other		PSMB6	proteasome (prosome, macropain) subunit, beta type, 6	peptidase	
MND1	meiotic nuclear divisions 1 homolog (S. cerevisiae)	other		SESN1	sestrin 1	other	
MPO	myeloperoxidase	enzyme	D, E, U	SLC30A7	solute carrier family 30 (zinc transporter), member 7	transporter	
MS4A3	membrane-spanning 4-domains, subfamily A, member 3 (hematopoietic cell-specific)	other		STK38	serine/threonine kinase 38	kinase	
NDC80	NDC80 kinetochore complex component	other		TMIE	transmembrane inner ear	other	
OLFM4	olfactomedin 4	other		YARS2	tyrosyl-tRNA synthetase 2, mitochondrial	enzyme	
RNASE2	ribonuclease, RNase A family, 2 (liver, eosinophil-derived neurotoxin)	enzyme	D	ZBTB10	zinc finger and BTB domain containing 10	other	
RNASE3	ribonuclease, RNase A family, 3	enzyme	E	ZNF384	zinc finger protein 384	transcription regulator	
SKA3	spindle and kinetochore associated complex subunit 3	other					
SPC25	SPC25, NDC80 kinetochore complex component	other					
STAR	steroidogenic acute regulatory protein	transporter					
TOP2A	topoisomerase (DNA) II alpha 170 kDa	enzyme	D, E, P, RT				
ZWINT	ZW10 interacting kinetochore protein	other					

#: D = diagnosis; DP = disease progression; P = prognosis; E = efficacy; RT = response to therapy; U = unspecified application.

^: Data resource: 2000–2014 Ingenuity Systems, Inc.

### LSC- 30-gene signature

**Figure 3:**
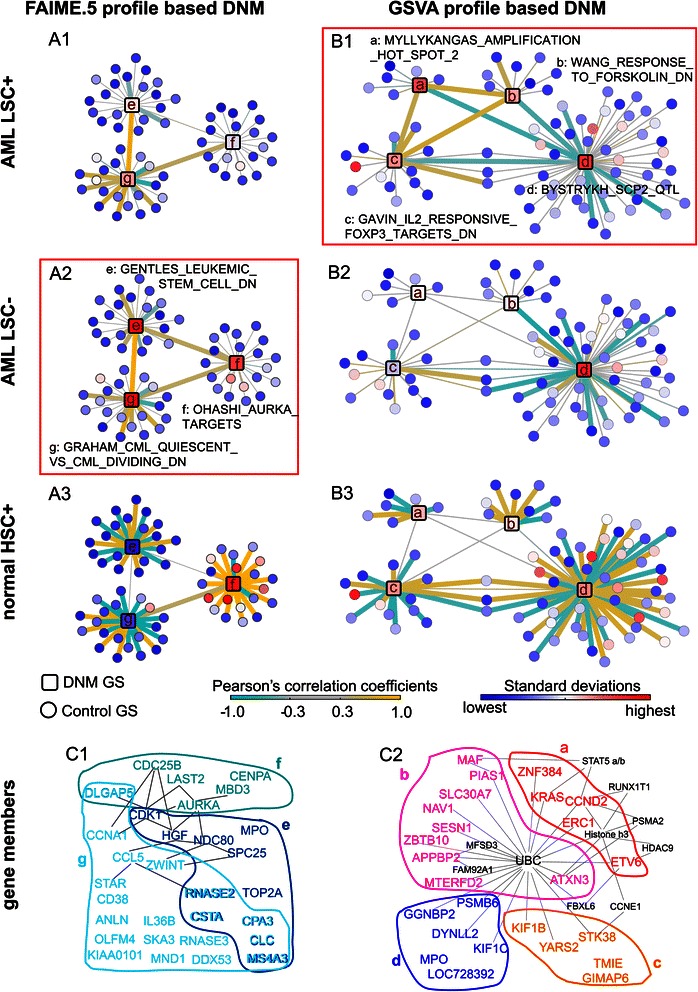
**Dynamic Network Mechanism (DNM) analysis on functional gene-sets (gene-sets).** Panel **A)** Network resulting from FAIME.5 profiles; Panel **B)** Network resulting from GSVA profiles. Each panel visually illustrates dynamics of the identified DNM gene-sets in each of the three sorted cell groups (1: AML LSC +, 2: AML LSC-, and 3: normal HSC+). Node color codes the standard deviation of a gene-set in the corresponding sample group, while line color codes the Pearson’s correlation coefficients between any two gene-sets. DNM gene-sets are represented as labeled squares and control gene-sets are represented as circles. The identified critical sample group for each analytical method is boxed in red (A1, B2). Line weight increases with correlation (>0.5 in Panel **A** and >0.4 in Panel **B**). Panel **C)** The colored groups of gene-members (a, b, c, d) or (e, f, g) corresponding to the above red-boxed DNM gene-sets respectively. The grey lines represent their pair-wise associations according to the Ingenuity knowledge database. Additional genes (black) that interacted with two or more identified genes in the Ingenuity database are also displayed.

Based on the FAIME.5 profiles, DNM identified 3 out of 3403 gene-sets representing expression signatures of chemical and genetic perturbations (**CGPs**) that distinguish LSC- cells from the other two cell subpopulations. In the LSC- population, genes in those three gene-sets exhibited different mutual correlations but lost many of their original correlated partners from the normal HSC+ population (Figure 3A), defining a new 30 member gene-set to represent LSC- exclusively.

Six (*DLGAP5, RNASE2, CSTA, CPA3, CLC, MS4A3*) out of the 30 genes shared significant overlap across the three gene-sets (p = 3.6e-15, Equation 7), indicating a functional cross-talk among the pre-defined gene-sets. Subsequent Ingenuity® connection analysis suggested that *CDK1* and *AURKA* play the role of ‘hub’ in this 30-genes network. For example, the 30 genes included six candidate substrate proteins (*MBD3, CDC25B, LATS2, DLGAP5, AURKA, CENPA*) of *AURKA* [25] (Figure 3A, node f). *AURKA* is a mitotic kinase over-expressed in AML CD34 (+) /CD38 (−) cells relative to their CD34 (+)/CD38 (+) counterparts or CD34 (+) normal HSCs [26]. Given the clinical impact of *AURKA* [26,27], the 30-gene signature reveals promising molecular targets to eliminate chemotherapy-resistance in LSC.

Also identified were two known gene-sets derived from previous studies about LSC stemness (Additional file 4: Table S2). One gene-set represents 11 genes (*SPC25, CPA3, NDC80, HGF, CSTA, CDK1, MS4A3, MPO, RNASE2, CLC, TOP2A*) down-regulated in the quiescent CD34+ cells when compared to dividing CD34+ cells isolated from the peripheral blood of myeloid leukemia patients [28]. The other gene-set contains 19 LSC down-regulated genes (e.g., *CD38, ZWINT, CCNA1*) when compared to leukemia progenitor cells from AML tumor samples [22].

### LSC+ 25-gene signature

The GSVA profiles identified four correlated gene-sets, consisting of 30 unique genes, that exclusively represent LSC+ cells (Figure 3B, Additional file 4: Table S3).

One DNM gene-set, stem cell proliferation-2 (Scp2) [29], included six genes (*DYNLL2, GGNBP2, KIF1C, MPO, PSMB6*, and *LOC728392*) that were physically mapped to the HSC proliferation quantitative trait locus (QTL) on chromosome 11 in mouse models (Figure 3B, node d). Scp2 is known to modulate the percentage of cells in S phase, and deletions of its corresponding region in human (maps to 5q31.1) have been associated with myelodysplastic syndrome and AML [29-31]. This gene-set is negatively correlated with the other three DNM gene-sets to represent LSC+ exclusively.

Another identified DNM gene-set contains five cancer genes (*CCND2, ERC1, KRAS, ZNF384, ETV6)* in the 12p13-p11.1 region with co-localized fragile sites (Figure 3B, node a). These amplified genes represent attractive targets for therapy, diagnostics, and prognostics [32]. This fact and the observation that 11 of these 25 genes are also significantly involved in cell death (*ATXN3, CCND2, ERC1, ETV6, KIF1B, KIF1C, KRAS, MAF, MPO, PIAS1, STK38*, p = 0.0068, Ingenuity pathway analysis - IPA, Ingenuity® Systems, www.ingenuity.com) indicate a clinical impact for prognosis.

Note that the DNM analysis on the gene-set level is designed to identify groups of genes with related functions and correlated expressions, rather than clustering samples on the gene-level. The expression changes of an identified gene might be subtle in one dataset or uncovered by its array. In fact, the joint gene-level expression pattern of the DNM identified gene-sets exhibits a correlation to data resources stronger than to cell types (Additional file 1: Figure S4, Additional file 5: Text S3). Regardless, the two identified gene clusters roughly clustered samples into two groups: LSC- samples or LSC+ samples. HSC+ samples are grouped together in each dataset. These results support the feasibility of using DNM analysis of an individual’s transcriptomic changes on a gene-set level to reveal functional biomarkers and biological underpinnings.

### Clinical relevance - prognosis of DNM gene-sets in patients with primary AML

To test the clinical relevance of the dynamic network mechanism analysis identified LSC representative gene-sets, we first investigated the gene-set profiles derived from patients with all types of AML in three independent cohorts (GSE14468, n = 518 [33]; TCGA, n = 197 [34]; GSE12417, n = 242 [35]). Across the three cohorts, increased scores of the identified gene-sets are associated with favorable overall survival, as log-regression coefficients of both clusters of DNM gene-sets are averagely negative (−0.16 and −0.02 respectively). We therefore hypothesized that the patients’ survival can be stratified by the identified DNM gene-sets and their selected control gene-sets that stand for tumor suppressor effects and tumorigenesis effects respectively. The controls are gene-sets that are significantly correlated with DNM gene-sets in normal HSC+ but not in LSCs cells, the circled nodes in Figure 3A-B.

To validate that hypothesis, we designed a novel Relative Effect Analysis with Gene-set Pairs (**RXA-GSP**) model, built from a parameter-free model that we have designed for personalized prognosis [17]. RXA-GSP calculates a prognostic indicator comparing scores of the identified gene-sets with scores of selected control gene-sets (Equation 8). For each identified gene-set-cluster and using the training cohort (GSE14468 [33]), we selected from all the control gene-sets a prognostic subset (p < 0.01, cox-regression coefficient > 0, Additional file 4: Tables S2, and S3). We then validated the indicator in two independent validation cohorts (TCGA [34]; GSE12417 [35]). Interestingly, a negative indicator significantly predicted a shorter survival in both training and the two validation cohorts. Additional file 1: Figure S5A shows the Kaplan-Meier plots of the indicator built from the gene-sets derived from FAIME.5 profiles (log-rank p = 0.00043, 4.7e-6, and 0.00032 respectively; empirical p = 0.007, 0.0045, and 0.007 by permuting random gene-sets with the same number of genes respectively). Note that the empirical p-value is more severe than the log-rank tested p-value, as random signatures might also predict cancer outcome [36]. This LSC-representative indicator remained independent of all known prognostic indicators including cytogenetic risk groups or European LeukemiaNet (ELN) risk groups, using multivariate analyses on overall survival of primary AML patients in all three cohorts (Table 3).

**Table 3:** **Univariate and multivariate analyses of overall survival in patients with all types of AML, for the LSC- DNM gene-sets**

**Dataset**	**Variate model**	**Variates**	**HR**	**95% CI**	**p-value**
**GSE14468 (n = 518)**	**Univariate model**	**3 DNM fGSs vs 4 control fGSs**	**0.7**	**0.55-0.84**	0.00043***
		ELN_RiskFavorable vs. Adverse	0.3	0.20-0.40	2.15E-13***
		complex vs. others	2.2	1.50-3.16	0.000024***
		7q vs. others	2.1	1.45-2.97	0.000051***
		ELN_RiskIntermediate-II vs. Adverse	0.5	0.36-0.70	0.000053***
		Age group, years (≥60 vs. <60)	1.7	1.29-2.28	0.00016***
		3q vs. others	2.1	1.32-3.42	0.0015**
		ELN_RiskIntermediate-I vs. Adverse	0.6	0.46-0.86	0.0035**
		inv16 vs. others	0.5	0.32-0.83	0.0057**
		cebpa mutation vs. others	0.6	0.67-0.95	0.028*
	**Multivariate model**	**3 DNM fGSs vs 4 control fGSs**	**0.7**	**0.60-0.91**	0.0050**
		Age group, years (≥60 vs. <60)	1.7	1.26-2.29	0.00046***
		ELN_RiskFavorable vs. Adverse	0.4	0.26-0.76	0.0034**
		complex vs. others	1.6	0.96-2.58	0.07.
		ELN_RiskIntermediate-II vs. Adverse	0.7	0.43-1.23	0.23
		7q vs. others	1.3	0.82-2.09	0.26
		3q vs. others	1.3	0.71-2.27	0.42
		inv16 vs. others	0.8	0.47-1.39	0.44
		cebpa mutation vs. others	0.9	0.53-1.45	0.61
		ELN_RiskIntermediate-I vs. Adver	0.9	0.53-1.48	0.65
**TCGA (n = 197)**	**Univariate model**	**3 DNM fGSs vs 4 control fGSs**	**0.46**	**0.32-0.64**	4.66E-06***
		Age group, years (≥60 vs. <60)	3.02	2.16-4.21	9.94E-12***
		gender	0.88	0.64-1.22	0.44
		normal_karyotype vs. others	1.12	0.81-1.55	0.50
		BM Blast(>50 vs. <=50)	0.88	0.60-1.30	0.53
	**Multivariate mode**	**3 DNM fGSs vs 4 control fGSs**	**0.60**	**0.42-0.86**	0.0058**
		Age group, years (≥60 vs. <60)	2.59	1.83-3.67	8.8E-08***
**GSE12417 (n = 242)**	**Univariate model**	**3 DNM fGSs vs 4 control fGSs**	**0.60**	**0.43-0.83**	0.0021**
		Age group, years (≥60 vs. <60)	1.63	1.18-2.26	0.0029**
	**Multivariate model**	**3 DNM fGSs vs 4 control fGSs**	**0.60**	**0.43-0.83**	0.0021**
		Age group, years (≥60 vs. <60)	1.49	1.07-2.07	0.018*

Significance code: ‘.’:p < .1; ‘*’: p < .05; ‘**’p < .01; ‘***’p < .001.

Significant univariate tested factors (p < .05) are used for multivariate test. Boldface highlights the results of DMN fGSs.

**Figure 4:**
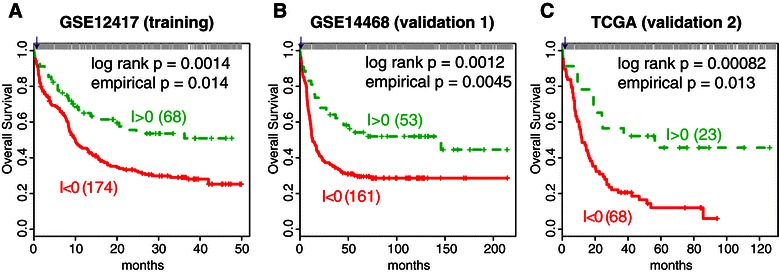
**Prognostic analysis of patients with cytogenetically normal AML, based on DNM identified gene-set pairs.** Kaplan–Meier plots of survival analysis on stratified samples with better outcome (green) or worse outcome (red). The Relative Effect Analysis with Gene-Set-Group Pairs (RXA-GSP) calculated a prognostic indicator by comparing three LSC- representative gene-sets (30 genes) with three normal control gene-sets (166 genes, Additional file 4: Table S2). The normalized FAIME.5 profiles are used. In each sub-panel, top bars mark the simulated p-values from which we estimated the empirical p-value for the actually observed log-rank p-value, the vertical line marked with an arrow. A RXA-GSP indicator I of less than 1 significantly indicates worse prognosis in the training cohort (GSE12417, Panel **A**) of cytogenetically normal AML patients and in two validation cohorts (GSE14468 and TCGA, Panels **B** and **C**) of cytogenetically normal AML patients.

Next, we investigated the clinical relevance in patients with cytogenetically normal AML. A subclass of intermediate-risk AML, cytogenetically normal AML has a variety of outcomes: some affected individuals respond well to standard treatment while others may require more intensive therapy. We set the largest cytogenetically normal AML cohort for training (GSE12417, n = 242 cytogenetically normal AML [35]), and the other two datasets as validation (GSE14468, n = 214 cytogenetically normal AML [33]; TCGA, n = 91 cytogenetically normal AML [34]). For the LSC- gene-sets, significance of three out of four of the control gene-sets was repeatable, based on the FAIME.5 profiles (Additional file 4: Table S2). The observed prognosis (log-rank p = 0.0014, 0.0012, and 0.00082 respectively; empirical p = 0.014, 0.0045, and 0.013, respectively, Figure 4) remains significant in multivariate analysis, independent of age, KRAS mutation, and ELN risk classification (Table 4).

**Table 4:** **Univariate and multivariate analyses of overall survival in patients with cytogenetically normal AML, for the LSC- DNM gene-sets**

**Dataset**	**Variate model**	**Variates**	**HR**	**95% CI**	**p-value**
**GSE12417 (n = 242)**	**Univariate model**	**3 DNM fGSs vs. 4 control fGSs**	**0.53**	**0.35-0.79**	0.0014**
		Age group, years (≥60 vs. <60)	1.63	1.18-2.26	0.0029**
	**Multivariate model**	**3 DNM fGSs vs. 4 control fGSs**	**0.6**	**0.37-0.83**	0.0038**
		Age group, years (≥60 vs. <60)	1.6	1.12-2.15	0.0083**
**GSE14468 (n = 214)**	**Univariate model**	**3 DNM fGSs v.s 4 control fGSs**	**0.5**	**0.33-0.77**	0.0012**
		KRAS mutaion vs. others	70.2	7.30-674.5	8.60E-13***
		ELN_risk (IntermediateI vs. Favorable)	1.8	1.27-2.62	0.00095***
		Age group, years (≥60 vs. <60)	1.4	0.89-2.18	0.15
		NPM1 mutation vs. others	0.8	0.57-1.12	0.19
		CEBPA mutation vs. others	0.7	0.42-1.22	0.22
		Gender	0.9	0.62-1.21	0.39
		BM Blast (>50 vs. <=50)	1.1	0.76-1.53	0.68
		NRAS mutation vs. others	1.0	0.60-1.81	0.90
	**Multivariate model**	**3 DNM fGSs v.s 4 control fGSs**	**0.5**	**0.35-0.83**	0.0047**
		KRAS mutaion vs. others	90.7	9.27-888.79	0.00011***
		ELN_risk (IntermediateI vs. Favorable)	1.7	1.19-2.48	0.0039**
**TCGA (n = 91)**	**Univariate model**	**3 DNM fGSs vs. 4 control fGSs**	**0.4**	**0.19-0.67**	8.23E-04***
		Age group, years (≥60 vs. <60)	2.5	1.58-4.09	6.53E-05***
		BM Blast (>50 vs. <=50)	0.6	0.33-1.09	0.09.
		Gender	0.7	0.46-1.16	0.18
	**Multivariate model**	**3 DNM fGSs vs. 4 control fGSs**	**0.4**	**0.23-0.84**	0.012*
		Age group, years (≥60 vs. <60)	2.1	1.32-3.47	0.0022**

Significance code: ‘.’:p < .1; ‘*’: p < .05; ‘**’p < .01; ‘***’p < .001.

Significant univariate tested factors (p < .05) are used for multivariate test. Boldface highlights the results of DMN fGSs.

Finally, we tested the relative effect analysis with gene-set pairs on the four gene-sets that were derived from the GSVA profiles to represent LSC+ cells. We identified a significant prognostic indicator by training the control gene-sets using all AML patients (Additional file 1: Figure S5B, log-rank p = 5.4e-5, 2.5e-5, and 0.05 respectively; empirical p = 0.002, 0.0005, and 0.15 respectively). The indicator is independent of all known clinic risk groups with an exception that it is dependent on patient ages bipartitely at 60 years old in the TCGA cohort (Additional file 4: Table S5). Strikingly, this cytogenetically normal AML indicator showed significance in two larger-sized validation datasets (Additional file 1: Figure S5C, log-rank p = 1.3e-10, p = 0.0002, and 0.05 respectively; empirical p < 0.001, 0.001, and 0.08 respectively).

Additionally, as a side-by-side comparison with gene-level signature, we tested the prognostic power of the “LSC signature”, the weighted sum of 31 genes defined by Gentles et al. [22]. In patients from GSE14468 and GSE12417, high LSC scores were associated with worse overall and event-free survival among patients with either normal karyotypes or chromosomal abnormalities (log-rank p < 0.002, Additional file 1: Figure S6 A1-A2, B), which is in agreement with previous publications. However, the patients in the TCGA cohort exhibit an exception. Their survival could not be predicted using the LSC score (Additional file 1: Figure S6 A3, C). One possible explanation would be, at least partly, the over-representation of mutations in the TCGA cohort (99% compared to the expected 75% in AML patients [37]). In contrast, our geneset-level indicators showed unified prognoses in all three tested cohorts.

### Biological relevance – Gene Ontology similarity, AML association, and correlation to RAS molecular activity

**Figure 5:**
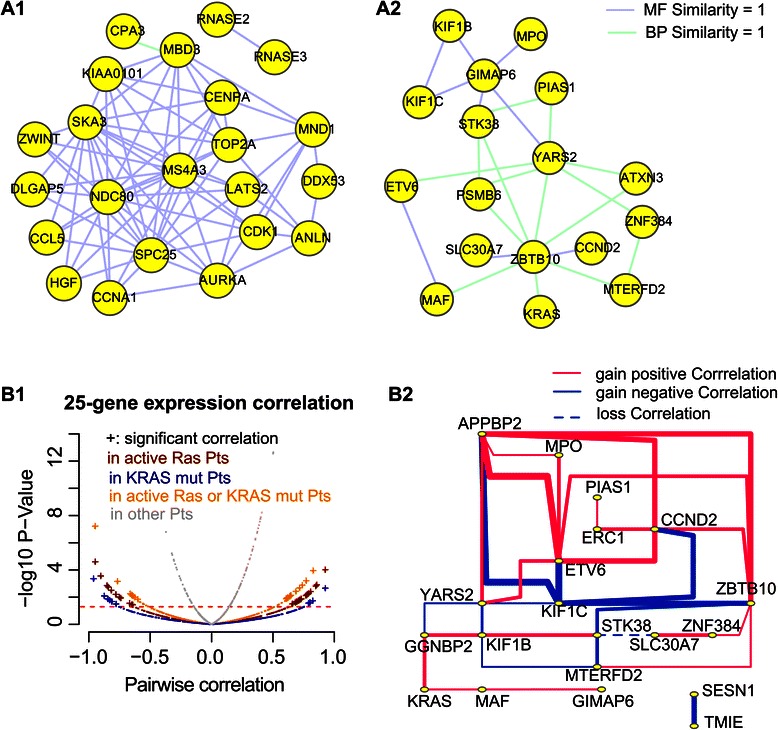
**The computationally evaluated biological relevance of genes in the identified gene-sets.** Panel **A)** Genes in the identified cluster of gene-sets share common functions. Sub-panel 1 represents the 30 LSC- representative genes and sub-panel 2 represents 25 LSC+ representative genes. Gene Ontology semantic similarity analysis reveals that 2/3 of the genes share the same function (the Lin distance =1). Line color codes the molecular function and biological process respectively. Panel **B)** Volcano plot (subpanel 1) of pairwise correlation tests between any two LSC+ 25-gene members (TCAG data). There are 31 significant co-expressions across 10 AML patients who show positive RAS activity (dark red). This co-expression disappears among the other 187 patients (grey), the 7 patients who carry somatic mutations of *KRAS* genes (blue), and the 16 patients carrying positive RAS activity or somatic RAS mutation (orange). Subpanel 2 illustrates the RAS activity-dependent co-expressed gene-pairs in a network. Comparing patients showing active molecular activity of RAS with patients showing normal RAS status, 31 pairs of genes gain significant co-expression, including 20 out of the 25 LSC+ representative genes (solid lines), whereas 1 gene-pair loses its co-expression in normal RAS status (the dashed line). Red lines represent positive correlations and blue lines represent negative correlations. Line widths correspond to Spearman correlation coefficients.

Gene Ontology (GO) provides curator-reviewed, standardized annotations for protein functions with a structured vocabulary. To evaluate co-functions of genes in the identified gene-set cluster, we employed semantic similarity of GO, a reliable computational method to exploit and classify coding gene functions (Additional file 2: Text S1: S Methods 2.4). For each DNM gene-set, two-thirds of the identified genes share molecular functional similarity or biological process similarity (semantic similarity score = 1, Figure 5A), explicating their shared dynamic phenotype in sorted cell populations and their consistent prognosis in primary AML samples. The 30 LSC- representative genes are intensively connected by 75 paired molecular functional similarity (empirical p = 6e-5, Figure 5A1), and the 25 LSC+ representative genes are intensively connected by 17 paired biological process similarity (empirical p = 0.03, Figure 5A2).

To further mine the biological and disease relevance of these gene-sets, we performed the Ingenuity pathway analysis (IPA) on the 30- and 25-gene sets respectively. Known cancer-related genes are over-represented in both: the LSC- 30-gene signature contains 22 cancer-associated genes (p = 0.0056), including three AML-associated genes (*AURKA, MPO, TOP2A*, p = 0.0048); the LSC+ 25-gene signature contains 10 cancer-associated genes, including another three AML-associated genes (*ETV6, KRAS, MPO*, p = 0.002). To interrogate novel AML-association in each signature, we re-ran IPA on the remaining LSC- representative (27-gene) subset and the remaining LSC+ representative (22-gene) subset excluding known AML genes. Interestingly, hematologic cancer genes (*CCL5, CD38, CDK1, HGF, IL36B, NDC80*, p = 0.0032) and genes linked with cell death of cancer cells (*CD38, CDC25B, CDK1*, p = 0.012) were overrepresented in the LSC- representative subset. Also, cancer cell transformation genes (*CCND2, MAF, ZNF384*, p = 0.018) and interphase of tumor cell lines (*CCND2, PIAS1, STK38, ZBTB10*, p = 0.000604) were enriched in the LSC+ representative subset. This result suggests a novel mechanism for AML tumorigenesis represented by these 30-gene and 25-gene signatures.

Finally, using available AML samples of the TCGA cohort [34], we investigated the relationship between the LSC+ 25-gene expression and the abnormal protein activity status of one gene member, *RAS*. The 25 genes gained co-expression (absolute Spearman coefficient >0.6 and p < 0.05) significantly among patients showing active RAS molecular activity (n = 10, empirical p = 0.048) but not among those carrying *KRAS* somatic mutation (n = 7, Additional file 4: Table S6) nor among those with normal RAS activity status (n = 187, Figure 5B).

We conclude that the LSC- signature of 30-gene or the LSC+ signature of 25-gene significantly share biological processes and molecular functions. Although mutations of RAS genes usually cause an intrinsic activation of RAS pathway in AML, it was RAS molecular activity, not genetic mutation, that perturbed the LSC+ signature of 25-gene from that of the control. The literature suggests in parallel that it was RAS molecular activity rather than its somatic mutations that exhibited a prognostic quality [38].

## Conclusion

### Computational strategies

Diverse signatures derived from the analysis of LSC gene expression profiles at the gene level confirm the heterogeneity of AML [5]. However, analyzing functional gene-sets can reveal common networks that are important for regulating LSC functions [39]. By meta-analysis and inter-dataset normalization, we have improved the reproducibility of characterizing clinically relevant LSC-signatures on the gene-set level. The other advantage of gene-set based algorithms is their ability to build functional profiles, facilitating computational identification and subsequent biological interpretation [7,21]. Building on gene-set-by-sample profiles, we successfully integrated microarray and RNA-seq data and performed two novel gene-set-analysis methods to reveal critical gene-sets for disease diagnosis and prognosis.

A precise gene-set-by-sample profile is a necessary prerequisite for functional class scoring approaches (reviewed by Khatri et al. [40]). It is the pan-genome weighting approach that more heavily weights highly-expressed genes and thus distinguishes FAIME.α from other gene-set analysis methods. Specifically, it biases of not only extremely high-valued genes by ranking but also the noise of low-valued genes by weighting, per sample. GSVA, on the other hand, heavily weights the two-tails of statistics and is sample-wise dependent [21]. FAIME.5 is an improved gene-set algorithm characterized by individualized*,* non-parametric, and un-supervised statistic (i.e., without the use of sample-wise estimation of differential expression).

We introduced novel dynamic network biomarker analysis on the gene-set level (DNM analysis) to represent LSC sub-populations. The identified gene-sets define new biomarkers for the regulation of stem cell function in AML, by characterizing dynamic features rather than the static differences. DNM has the ability to discovery significant regulatory changes of functional gene-sets across disease progression stages. By taking into account of a critical transition from normal state to disease state during cell development, DNM outperforms other gene-set analytic strategies in capturing critical signatures. The proposed RXA-GSP (Relative Effect Analysis with Functional gene-set-Group Pairs algorithm) is a parameter-free model with respect to gene-sets and is designed to bridge cancer biology from the lab to the clinic [17,18]. It can be extended to other applications when analyzing biological imbalance within a patient. RXA-GSP naturally fits the “two-hit hypothesis” for malignancy: one leads to uncontrolled cellular proliferation and evasion of apoptosis and the other adjusts inhibition of differentiation [41]. It is particularly useful for efficiently translating microarray or RNA-seq data to clinical discoveries.

Altogether, researchers can apply these proposed computational strategies to study other diseases in a systematic “gene-to-function, snapshot-to-dynamics, and biology-to-clinic” framework. Previously, we have successfully demonstrated the application of gene-to-function (FAIME) on head and neck cancer, snapshot-to-dynamics (DNB) on diabetes, and biology-to-clinic (RXA-GSP) on breast cancer [7,16,17]. Increasing evidence suggests the feasibility of analyzing an individual’s transcriptome on a pathway-level for clinical decision-making and precise mechanism comprehension. Additionally, different methods perform differently under different conditions or parameter settings in their ability to detect the complex abnormality in cancer. Researchers need to make ad hoc modifications and select among analysis algorithms to make biological discoveries.

### Biological discoveries

This work sheds promising insight into gene-set profiles by defining the LSC- signature of 30-gene and the LSC+ signature of 25-gene for prognosis in AML. These signatures suggest potential therapeutic regimens to eradicate quiescent, chemotherapy-resistant LSCs, because they exhibit two key characterizes - independence of cell cycle status and not substrates for drug efflux pump proteins [39]. The LSC- signature of 30-gene exposed a promising molecular target, *AURKA* [26,27], to potentially eliminate chemotherapy-resistance in LSC. The LSC+ signature of 25-gene included known cancer genes in the 12p13-p11.1 region [32] as well as genes in the 5q31.1 region [29] that are known to be associated with myelodysplastic syndrome and AML. The only common gene from both signatures, myeloperoxidase (*MPO*), is implicated as a biomarker for favorable prognosis in AML and its transcription levels reflect epigenetic modification [42]. This is not surprising because *MPO* is known to be expressed by the myeloid lineage in AML but not in HSC [43]. Given the complex system changes in tumor progression, we expect that new gene-set clusters could be prioritized using alternative GSEA methods.

The two identified clusters of gene-sets significantly predicted overall survival for 1478 primary AML patients, regardless of the inter-patient and intra-patient variability of AML phenotypes. Their prognostic independence is consistent with the published LSC-derived prognostic signature. However, previously identified genes are associated with adverse prognosis in AML [22]. Our results suggest that the regulation of stem cell function in AML also contains a favorable prognostic component. Notably, the ability of LSC- representative gene-sets to significantly stratify 548 patients with cytogenetically normal AML is intriguing for precision medicine, as cytogenetically normal AML is difficult to stratify for patient outcomes. Evidence from the literature further supports clinical relevance of the identified DNM gene-sets and the selected control gene-sets. These LSC- control gene-sets included published LSC highly-expressed genes that were previously associated with unfavorable event-free survival in patients with AML [22]. The LSC+ control gene-sets included fenretinide-down-regulated genes that were significantly correlated with poor-prognosis in AML patients [44]. In contrast, the LSC- DNM gene-sets are down-expressed in LSC and consist of genes indicating good outcome (and/or) genes being repressed by forskolin or IL2, two AML drugs that stop blood cell differentiation or induce prolonged remissions in advanced AML [45]. Genes associated with the Scp2 GWAS study [29] are potential new drug targets according to our results.

Genes from the identified functional gene-sets significantly share biological processes and molecular functions, suggesting a new aspect of stem cell biology. The questions of how these genes coordinate in the regulation of stem cell function or determine “stemness” warrant future investigation. We confirmed the biological co-expression of the LSC+ signature of 25-gene in AML patients carrying positive RAS molecular activity. As previous publications attest, it was RAS molecular activity rather than gene mutation that disturbed the identified LSC+ signaling and showed a prognostic factor.

In conclusion, LSC, or leukemia-initiating cell is a rare cellular subpopulation that must be eradicated to cure a patient of leukemia. However, their underlying mechanisms remain a biological conundrum, partly due to limited sample size and inter-patient and intra-patient variability. This study proposes a comprehensive knowledge-driven systematic analysis to functionally characterize LSC collected by different laboratories, followed by a novel dynamic network analysis. Two identified LSC- subpopulation-specific gene-set clusters, showing significant biological and clinical relevance, have been validated independently. The proposed framework extends our ability to re-use multiple layers of “omics”-data, to derive a new gene-set from coordinated gene-sets, and to discover new prognostic indicators, thus bridging cancer biology in the lab and the clinic.

## Methods

### Data

#### Patient samples

All datasets were previously published (Additional file 4: Table S4) and are publicly available. We have additionally received written authorization from the authors of GSE14468 to re-use the survival time of their samples. All samples of patients with AML were obtained at the time of diagnosis and with informed consent at corresponding hospitals, and study protocols were approved by the institutional review boards of corresponding institutes and hospitals [34,35,46,47].

#### Gene expression

We performed a literature review from PubMed, GEO, and ArrayExpress for three keywords (“stem cell”, “AML”, and “prognosis”) in October 2012. We collected nine LSC studies (Table 1 and Additional file 4: Table S1), two large primary AML datasets [35,46] (n > 200 each) and samples from TCGA [34] (Additional file 4: Table S4). We pooled sorted cell samples into three groups defined by cell surface markers: AML LSC-enriched cells (LSC+), AML LSC-depleted cells (LSC-), and normal samples of sorted HSC-enriched cells (HSC+). Only functionally defined LSC+ samples (n = 77) were investigated, showing *in vivo* validated leukemia stemness in xenograft models. The HSC+ samples (n = 23) included diverse microarrays and RNA-seq measurements (Table 1).

#### Functional gene-set

We studied three categories of previously defined functional gene-sets from MSigDB (version 4.0) [19], with variable numbers of member genes (Additional file 2: Text S1: S Methods 2.1).

### Functional gene-set profiles

#### Collapse multiple measurements per gene

To convert the gene-by-sample values into the gene-set-by-sample scores, we first collapsed the gene expression profiles to one value per sample for each unique gene, using a selective collapsing strategy with the highest average expression. This strategy has previously led to the best inter-study consistency [48]. This step is necessary to ensure equal gene representation as some genes have multiple measurements on a microarray.

#### Calculate gene-set profiles

We calculated the FAIME-scores [7], the GSVA-scores [21], and the new FAIME.α-scores using Equations 1, 2, 3, from all genes for each sample (*j*) (hereafter referred to as the “gene-set profile”).

The null statistical hypothesis of a FAIME score is that the weighted ranks of genes inside a gene-set and outside the gene-set are drawn from the same distribution. In our calculations, we first converted the original gene expression values to ranks (*r (x)*_*j*_) to reduce the influence of potential outliers. Then we converted the ranks per sample into a new scale (*s (x)*_*j*_) of continuous values to softly award highly-expressed genes [20]. FAIME.5 employs qualitative scores sharply decreasing along gene ranks by assigning the parameter α = 5 (Equation 1). This strategy filters out low expressed genes and maintains cohort-independence.

Let *i* denote the gene-set index, by *j* the sample index, *x* the gene index, and *n* the total number of measured genes. Then

1 $$ s{\left(x,\alpha \right)}_j=r{(x)}_j\cdot w\left(x,\alpha \right)\kern0.24em \mathrm{where}\kern0.24em w\left(x,\alpha \right)={e}^{\alpha \left(\frac{r{(x)}_j}{n}-1\right)} $$

where the parameter *α* controls the rate at which the weights rise when moving the list from the lowest expression ranks to the highest. A sequence of rapidly increased weights (large *α*) is sensitive to the genes at the extreme top of expression ranks (Figure 2B). Our previous research has shown that the ability to discriminate between signal and noise critically depends on the original dataset [14], and a value of *α =* 1 was used in previous data [7].

Given a set of genes {*x*_*1*_ *,…,x*_*m*_}∈ *gene-set*_*i*_ of size *|gene-set*_*i*_ *|*, we scored its activity function *A()* using Equation 2 and that for its complementary set ${\overline{GS}}_i$.

2 $$ A{\left(x\in fG{S}_i,\alpha \right)}_j=\frac{1}{\left|fG{S}_i\right|}{\displaystyle \sum_{x\in G{S}_i}s{\left(x,\alpha \right)}_j}\begin{array}{cc}\hfill; \hfill & \hfill \hfill \end{array}A{\left(x\notin fG{S}_i,\alpha \right)}_j=\frac{1}{\left|{\overline{fGS}}_i\right|}{\displaystyle \sum_{x\notin G{S}_i}s{\left(x,\alpha \right)}_j} $$

Equation 3 defines a gene-set score which is the difference between the activity function of genes inside and outside a previously defined gene-set.

3 $$ FAIM{E}_{i,j}\left(\alpha \right)=A{\left(x\in fG{S}_i,\alpha \right)}_j-A{\left(x\notin fG{S}_i,\alpha \right)}_j $$

#### Inter-dataset normalization

The goal of normalization was to compensate for technical differences and thus to make gene-set profiles from different resources comparable [49,50]. Inter-dataset normalization is implemented using the z-transform on the gene-set profile *f.*_*j*_ = {*f*_*1*_ *,f*_*2*_ *,…f*_*N*_} per sample *j* by dividing by the standard deviation of the centered values, which is denoted *y.*_*j*_ in Equation 4. *Y* is a combined matrix where rows correspond to pathways and columns correspond to samples. This type of standardization has been applied to two types of analyses: integrating gene-set scores of differentially expressed genes and analyzing trait-associated genetic markers [13].

4 $$ Y=\left\{{y}_{\cdot j}\right\}=\frac{f_i-\mu }{\sigma \left({f}_i-\mu \right)}\kern0.24em \mathrm{where}\kern0.24em \sigma (.)=\sqrt{\frac{1}{N}{\displaystyle \sum_{i=1}^N{\left({f}_i-\mu \right)}^2}},\mu =\frac{1}{N}\left({f}_1+\dots +{f}_N\right) $$

### Unbiased simulation

To evaluate statistical power (sensitivity) and type-I error, we carried out a simulation study as previously described (Additional file 1: Figure S1) [21]. In short, normalized and log-transformed gene expression values were simulated for each gene *a* = {1,2,…*p*} and sample-group *b* = {1,2} using the linear additive model in Equation 5,

5 $$ {y}_{ab}={\alpha}_a+{\beta}_b+{e}_{ab} $$

where gene specific effect is *α*_*α*_ ~ *N*(0,1), sample specific effect is *β*_*b*_ *~ N(μ*_*j*_ *,σ*_*j*_ *)*, and random noise is *e*_*iab*_ *~ N*(0,1).

In the simulation study, we modeled *p* = 5000 genes and two groups of samples each with different sample size n = {10, 20, 40, 60, 80, 100}. We randomly built a differentially expressed (**DE**) gene-set of 30 differentially-expressed genes and a non-DE gene-set of 30 no-changed genes, considering the following two facts for the DE gene-set. 1) The fraction of differentially expressed genes in the DE set varied, 50% and 80% respectively. 2) The expected signal-to-noise-ratio varied between weak and strong, meaning that the magnitude of DE set between two sample groups was 0.5 and 1. The simulation was done by setting *μ*_*1*_ *= μ*_*2*_ = 0 with *σ*_1_ *= σ*_2_ = 1 for all genes in the non-DE set but *μ*_1_ = m, *μ*2 = 0 with *σ*_1_ *= σ*_2_ = 0.5 for a certain fraction of genes in the DE set. Then we applied GSEA, GSVA, and FAIME modulating the parameter *α* = {1, 5, 10} (Equations 1, 2, 3) on the simulated gene expression data to generate the simulated gene-set scores respectively. We repeated the above simulations assigning the DE and non-DE gene-sets with *x* = {10, 20, 80, and 100}, i.e., 0.2%, 0.4%, 1.6%, and 2% of the modeled 5000 genes. The statistical power was a fraction of significance for the DE set (true positive) whereas the empirical type-I error was a fraction of the non-adjusted significance (p < 0.05, two-sample t-test) of the non-DE set. We adjusted the p-values for multiple-testing across 1000 simulations [51] and set a significance level of FDR = 0.05.

### Dynamic Network Mechanism (DNM) analysis

DNM is built on dynamic network biomarker analysis (DNB) [15], a model-free method to detect the dynamics of disease developmental stage changes using gene expression profiles. Developed for gene-set profiling, DNM identifies a cluster of gene-sets that highly co-fluctuate in a “critical stage” (or disease transition stage). It explores the dynamic character of network nodes (gene-sets) and links (alteration between gene-set pairs) across sample groups. Given a sample group *t* = {LSC+, LSC-, and normal HSC+} and the assigned control group (*e.g.,* the normal HSC+), we performed the following five-step calculation in our DNM analysis Figure 3.

1. To acquire candidate gene-sets with the highest intra-group variation, we calculated the standard deviation (SD) of every gene-set across samples within this sample group. Then, we picked *gene-set*_*t*_, the top 5% of all gene-sets having the highest SDs.
2. To cluster the *gene-set*_*t*_ into highly co-variable modules in a sample group *t* of interest, termed *C*_*t*_ *I*, we calculated the ‘intra-module’ correlation of SDs (gene-sets were compared in pairs). We ran an unsupervised hierarchical clustering on overall pairwise Pearson correlation coefficients (PCC). This agglomerative hierarchical clustering was performed in R using complete linkage on the pairwise PCC distance (1-PCC) in *gene-set*_*t*_. A module of *gene-set*_*t*_ consists of gene-sets that satisfy a significant level (p-value ≤ 0.01). Applying a threshold of p-value ≤ 0.001 or p-value ≤ 0.05 also leads to comparable DNMs.
3. To estimate ‘inter-module’ co-variation within this sample group *t* (*C*_*t*_ *I*), we calculated the correlation of SDs between *C*_*t*_ *I* members and other control gene-sets. We also estimated ‘cross-module’ co-variation between this sample group t and control group (*C*_*t*_ *O*). The control gene-sets preserved the top n highest correlations with any *C*_*t*_ *I* members in the control group but outside the *C*_*t*_ *I* set. We applied n = 5 to small- or middle-sized gene-sets (pathways and motifs) and n = 20 to the larger-sized gene-sets (the CGPs).
4. To determine the critical sample group, we calculated a combination score *S*_*t*_ *I* in Equation 6 [15] for each module *C*_*t*_ *I* in a given sample group *t*.

6 $$ {S}_tI=\frac{S{D}_{tI}\cdot PC{C}_I}{PC{C}_o} $$

where *SD*_*tI*_ is the average SD value of gene-sets in the *C*_*t*_ *I*, *PCC*_*I*_ represents the average PCC value of gene-set-pairs in the *C*_*t*_ *I*, and *PCC*_*o*_ the average PCC value of gene-sets between this *C*_*t*_ *I* and *C*_*t*_ *O*. The *C*_*t*_ *I* with the largest *S*_*t*_ *I* score was then determined to comprise the DNM markers and the corresponding sample group *t* to be the critical sample group (disease stage).

5) Additionally, to assess the significance of gene overlap among DNM gene-sets for a critical sample group, we calculated the hypergeometric probability using Equation 7 (Additional file 2: Text S1: S Methods for inference). Given A, B, and C to be the number of gene-set size for three DNM gene-sets respectively, N = 22000 the background number of human genes, *n* the observed number of shared genes among DNM gene-sets, we calculated:

7 $$ p\left(x=n\Big|A,B,C,N\right)={\displaystyle \sum_{k=0}^n\begin{array}{ccc}\hfill \frac{\left(\begin{array}{c}\hfill A\hfill \\ {}\hfill k\hfill \end{array}\right)\left(\begin{array}{c}\hfill N-A\hfill \\ {}\hfill B-k\hfill \end{array}\right)}{\left(\begin{array}{c}\hfill N\hfill \\ {}\hfill B\hfill \end{array}\right)}\hfill & \hfill \cdot \hfill & \hfill \frac{\left(\begin{array}{c}\hfill A+B-2k\hfill \\ {}\hfill n-k\hfill \end{array}\right)\left(\begin{array}{c}\hfill N-A-B+k\hfill \\ {}\hfill C-n+k\hfill \end{array}\right)}{\left(\begin{array}{c}\hfill N\hfill \\ {}\hfill C\hfill \end{array}\right)}\hfill \end{array}} $$

### Prognosis of DNM gene-sets on primary AML samples

Previously, we proposed the concept of relative-expression of gene-set pairs [17] which calculates a prognostic indicator (Equation 8) for each patient. An indicator of Relative Effect Analysis with Gene-set Pairs (RXA-GSP) compares effects of DNM gene-sets with selected control gene-sets, instead of genes, for each patient.

8 $$ {I}_j\left(S,C\right)=\left({f}_j(S)-{f}_j(C)\right)=\left\{\begin{array}{c}\hfill >0\  good. outcome\hfill \\ {}\hfill <0\  poor. outcome\hfill \end{array}\right. $$

Based on the DNM analysis, we selected control gene-sets {C} meeting the following two criteria: 1) significant univariate analyses on overall survival of AML patients (log-rank p < 0.01), and 2) a positive cox-regression coefficient (*i.e.*, increased scores of selected control gene-sets are associated with poor outcome). We then derived a sum-value *S*_*j*_ in Equation 9 for the DNM gene-set group {S} and the control gene-set group {C}, respectively. The sum-value is a linear combination of the gene-set scores (indexed by *i*) weighted by the respective gene-set’ cox-regression coefficient in the training set [47].

9 $$ {f}_j(S)={\displaystyle \sum_{i\in S}coe{f}_{ij}\cdot FAIM{E}_{ij}\begin{array}{cc}\hfill; \hfill & \hfill {f}_j(C)={\displaystyle \sum_{i\in C}coe{f}_{ij}\cdot FAIM{E}_{ij}}\hfill \end{array}} $$

Gene-set scores of all identified DNM gene-sets and their corresponding control gene-sets were calculated for primary AML patients in three independent cohorts to identify an indicator (Additional file 4: Table S4). We set the largest cohort as the training set and used the other two cohorts for validation. The treatment-related AML patients and patients with prior malignancy or leukemogenic agent exposure were excluded.

The Kaplan-Meier and the univariate Cox regression analyses on both the training set and validation set demonstrated the prognosticative power of the indicator. We then asked whether the novel indicator remained prognostic after adjusting by all other significant variables that had a univariate p < 0.05, using multivariate testing.

Additionally, an empirical evaluation was performed as suggested [52], by replacing the FAIME scores in Equation 8 with randomly picked |S| and |C| gene-sets from 3420 CGPs in the MSigDB database and repeating the above two calculations *X* = 2000 times, where the cardinality of a set S is denoted |S|. Then we estimated the chance of observing the observed log-rank p-values using Equation 10, the fraction of simulated p-values that are less than or equal to the observed p-values.

10 $$ p=\left|p{\hbox{'}}_{\log - rank}\le {p}_{observed}\right|/X $$

## Additional files

Additional file 1: Figure S1. Statistical power and type-I error rate in unbiased simulations. **Figure S2.** Published LSC associated gene signatures and their significantly enriched canonical pathways. **Figure S3.** Distribution of pathway profiles for collected samples. **Figure S4.** Heatmap of genes in the two DNM identified gene clusters from different datasets of three cell subpopulations. **Figure S5.** Kaplan-Meier plots of patients with primary AML, based on DNM identified gene-set pairs. **Figure S6.** Kaplan-Meier plots of patients with primary AML, based on Gentles “LSC signature” [22].
Additional file 2:
**Text S1.** Supplementary results, methods, and the limitation.
Additional file 3:
**Text S2.** R Code and demonstration for the function rungene2pathway.
Additional file 4: Table S1. Nine studies and eight published gene lists pertaining to AML LSC. **Table S2.** LSC- representative DNM gene-sets (30-gene) and their control pairs in the normal state, derived from gene-set profiles using the FAIME.5 algorithm. **Table S3.** LSC+ representative DNM gene-sets (25-gene) and their control pairs in the normal states, derived from gene-set profiles using the GSVA algorithm. **Table S4.** Three independent primary AML studies. **Table S5.** Univariate and multivariate analyses of overall survival in patients with all types of AML, for the LSC+ representative DNM gene-sets. **Table S6.** The somatic mutations (of genes in the identified DNM gene-sets) and their patient information in the TCGA database.
Additional file 5:
**Text S3.** A tab-delimited text file recording the normalized expression of the 54 identified genes.

## Abbreviations

AML: Acute myeloid leukemia
CGP: Chemical and genetic perturbations
C_t_I: ‘Inter-module’ co-variation within this sample group *t*
C_t_O: ‘Cross-module’ co-variation between this sample group *t* and control group
DNB: Dynamic network biomarker
DNM: Dynamic network mechanism
SD: Standard deviation
ELN: European LeukemiaNet
FAIME: Functional analysis of individual microarray (or RNA-seq) expression
FDR: False discovery rate
IPA: Ingenuity pathway analysis
GO: Gene ontology
GS: Gene set
GSEA: Gene set enrichment analysis
GSVA: Gene set variation analysis
HSC+: Verified hematopoietic stem cells enriched
LSC: Leukemic stem cell
LSC+: LSC enriched
LSC-: LSC depleted
MSigDB: Molecular signature database
PCC: Pearson correlation coefficients
RXA-GSP: Relative effect analysis with gene-set pairs
