# Supplementary material for: Systematic computation with functional gene-sets among leukemic and hematopoietic stem cells reveals a favorable prognostic signature for acute myeloid leukemia
Source: BMC Bioinformatics. 2015 Mar 24;16:97. doi: 10.1186/s12859-015-0510-7 (PMC4376348; doi:10.1186/s12859-015-0510-7)
Supplement: Additional file 1: Figure S1. — Statistical power and type-I error rate in unbiased simulations. Figure S2. Published LSC associated gene signatures and their significantly enriched canonical pathways. Figure S3. Distribution of pathway profiles for collected samples. Figure S4. Heatmap of genes in the two DNM identified gene clusters from different datasets of three cell subpopulations. Figure S5. Kaplan-Meier plots of patients with primary AML, based on DNM identified gene-set pairs. Figure S6. Kaplan-Meier plots of patients with primary AML, based on Gentles “LSC signature” [22]. [file 12859_2015_510_MOESM1_ESM.pdf]

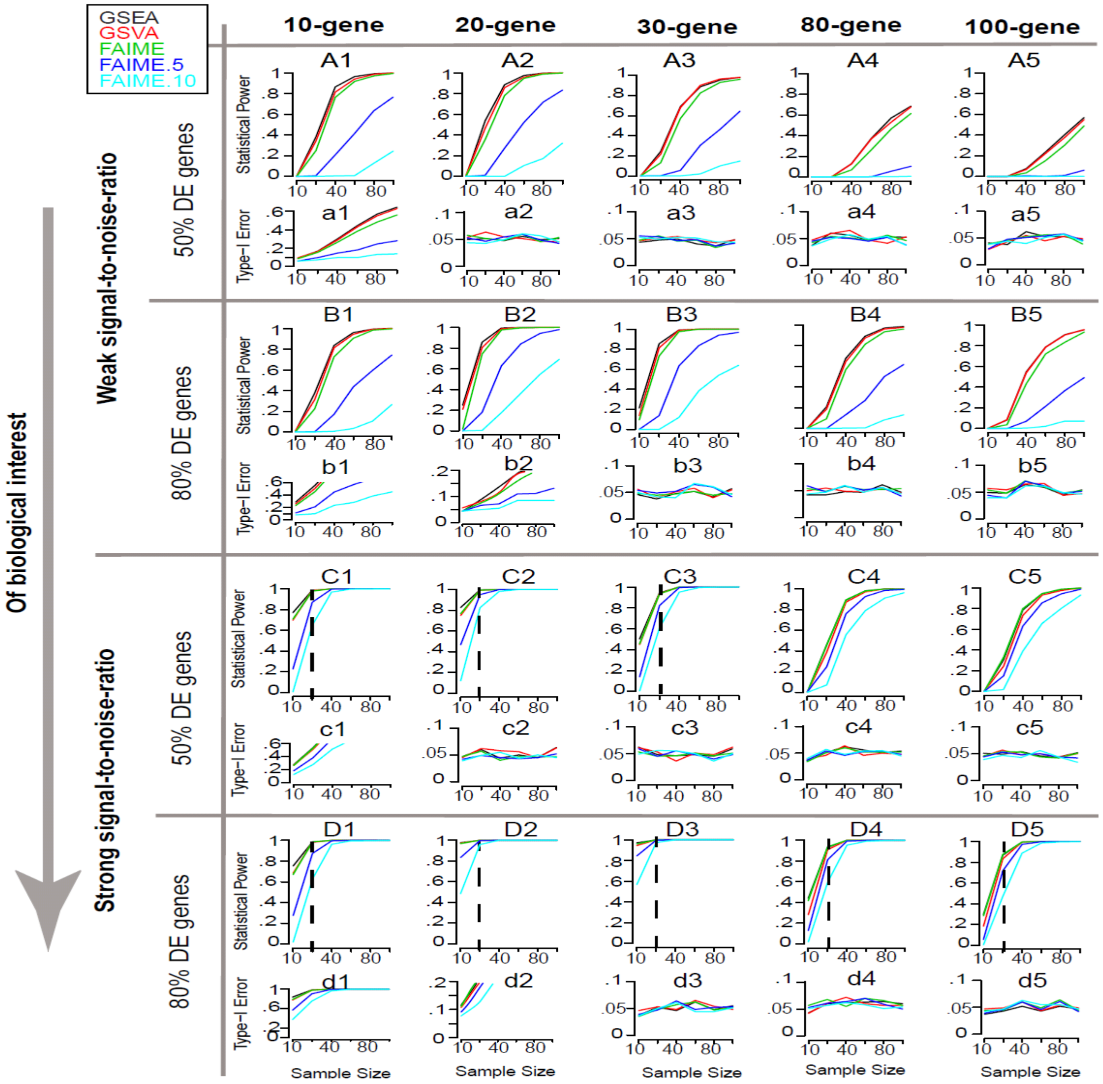

Figure S1. Statistical power and type-I error rate in unbiased simulations. Results based on GSEA, GSVA and FAIME (with different parameter *α* settings) were color coded. The respective statistics (y-axis) were estimated by a t-test on simulated differentially expressed (DE) and non-DE gene-sets respectively, using several sample sizes (x-axis). The DE and non-DE gene-sets were simulated from a linear additive model for 5,000 genes as previously described (BMC Bioinformatics 2013, 14:7). Functional gene-sets scores were then calculated with each method.

Four scenarios were simulated: **A-a**) weak signal-to-noise ratio, 50% of DE genes in the DE set; **B-b**) weak signal-to-noise ratio, 80% of DE genes in the DE set; **C-c**) strong signal-to-noise ratio, 50% of DE genes in the DE set; **D-d**) strong signal-to-noise ratio, 80% of DE genes in the DE gene set.

In each simulation scenario, the results for simulated gene-sets with different gene sizes (x=10, 20, 30, 80, 100) are shown. The corresponding top subpanel (**A, B, C, D**) depicts the averaged statistical power at a significance level of FDR=0.05, and the bottom subpanel (**a, b, c, d**) gives the type-I error rate at a significance level of unadjusted p=0.05 given a gene-set size and after 1,000 simulations in each scenario. The dashed line indicates the scenario in which a higher than 80% accuracy is achieved using a sample size of 20.

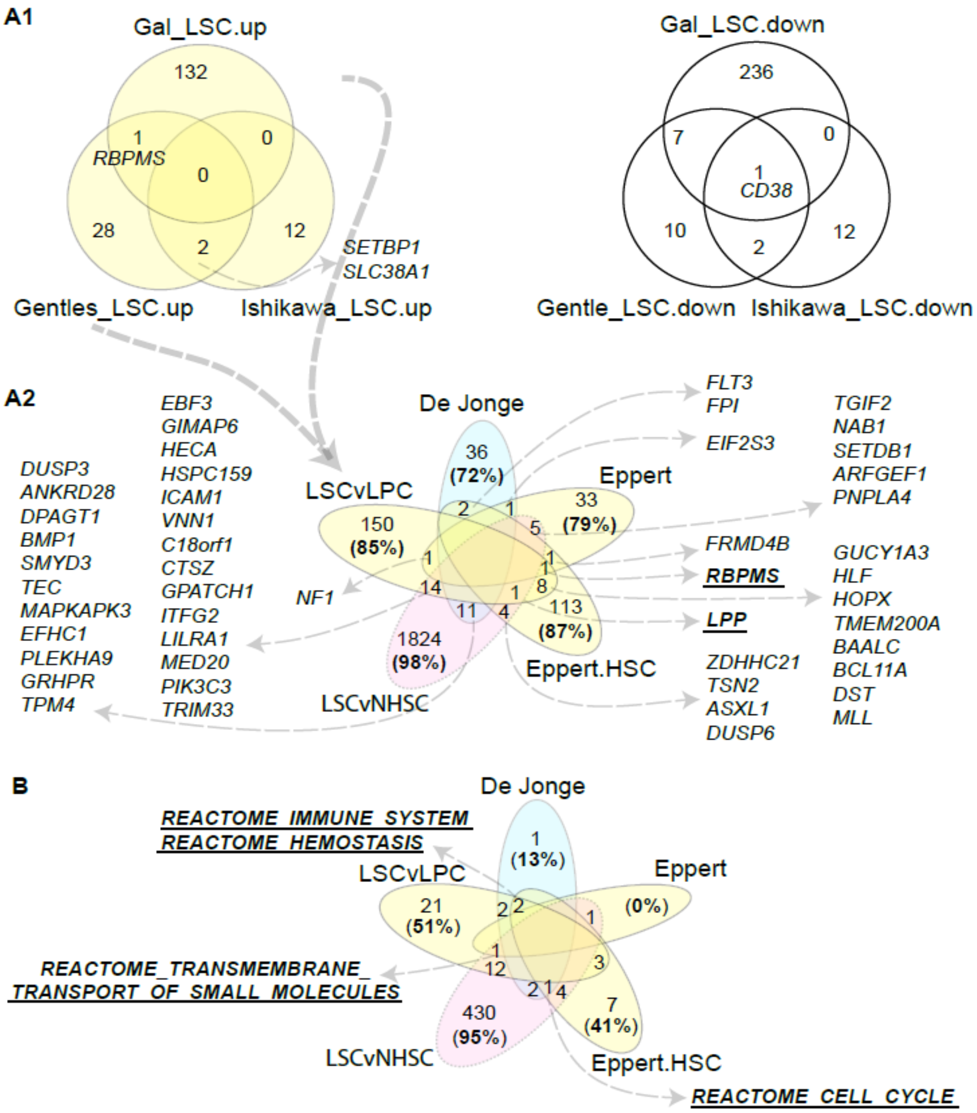

Figure S2. **Published LSC associated gene signatures and their significantly enriched canonical pathways**.

A1) Low overlap among gene signatures in three independent studies to distinguish LSC-enriched cell populations (CD34+CD38-) from more mature leukemia progenitor cell (LPC) populations (CD34+CD38+), purified from the same AML patient. We merged these three LSC highly expressed gene-lists and refer to them as “LSCvLPC” hereafter. **A2)** Overlap among five LSC highly expressed gene signatures in three categories: AML stemness (yellow), AML LSC-specific (blue), and AML malignance (pink).

B) Canonical pathway overlap among enrichment analysis results of the above five gene signatures (FET p<0.05, gene count≥3). The functional-level enrichment analysis signatures have remarkably higher cross-study repeatability when compared with gene-level signatures. In both Panels A2 and B, the percentage of dataset-unique signature is given for each gene-list.

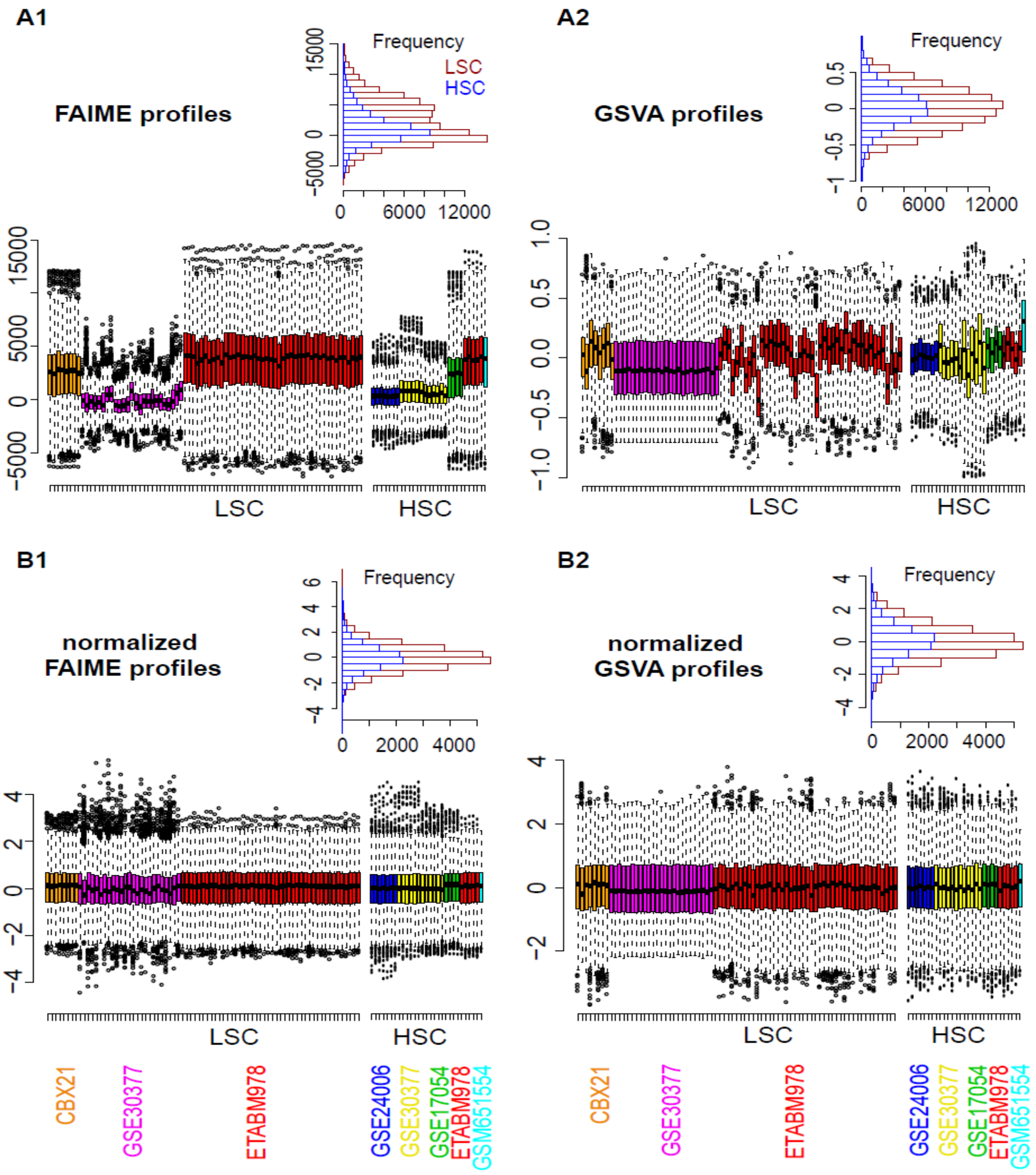

Figure S3. **Distribution of pathway profiles for collected samples**. Canonical pathway profiles using the FAIME method before and after normalization (**Panels A1 & B1** respectively). Pathway profiles calculated by the GSVA method before and after normalization (**Panels A2 & B2** respectively).

In each panel, we summarize the gene-set score distribution in a histogram for 106 collected samples and a combined sample box-and-whisker plot. Histograms represent the overall data distribution of LSC (red) and HSC (blue). Box-and-whisker plots represent individual sample distributions that are categorized as LSC+ (left) or HSC+ (right). Color coded dataset resource is displayed at the bottom of the figure. The central box represents the values of a function profile (y-axis) from the lower to upper quartile. The middle line represents the median. The horizontal line extends from the minimum to the maximum value within 1.5 times of the interquartile range from the box.

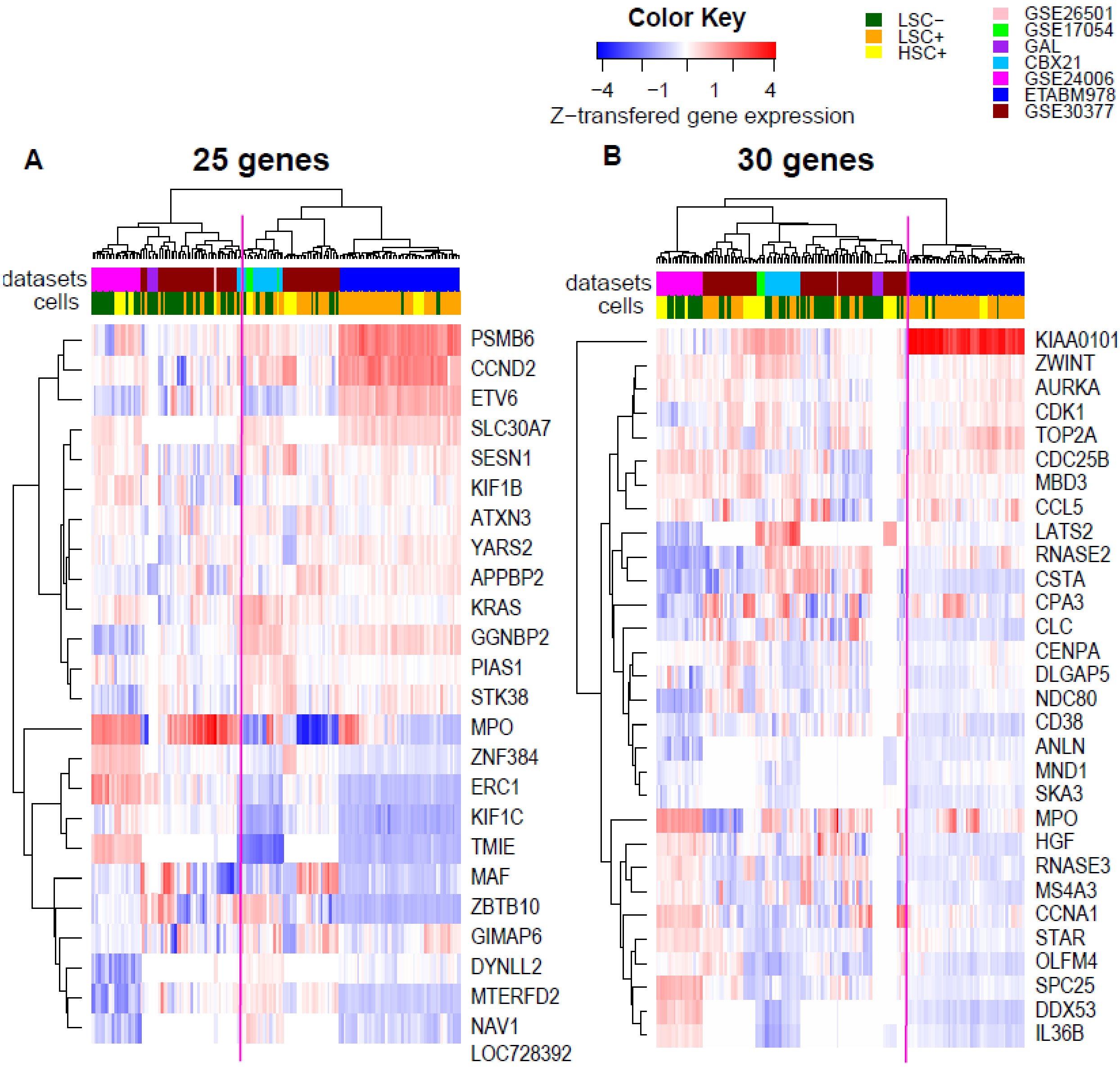

Figure S4. Heatmap of genes in the two DNM identified gene clusters from different datasets of three cell subpopulations. Each heatmap illustrates one sample per column using the ward.D2 hierarchical clustering algorithm and Euclidean distance metrics (R stats package). Red indicates relative high expression, blue indicates relative low expression, and white indicates the NA or zero values. The two top colored bars indicate the sample resources and cell sub-populations. The previously normalized gene expression values were z-transferred (per sample) to allow cross-dataset comparison, and the results illustrate that the major samples from the same dataset are clustered together. Note that too many NA values ((>10% of gene-to-sample values across seven datasets using different technologies) restricted the analysis on the gene-level directly. Regardless of these barriers, the two identified gene clusters roughly clustered samples into two groups (by the vertical red line, respectively): LSC- samples or LSC+ samples. HSC+ samples are grouped together in each datasets. These results support the feasibility of analyzing an individual's transcriptomic changes on a gene-set-level to reveal the functional biomarkers and biological underpinnings.

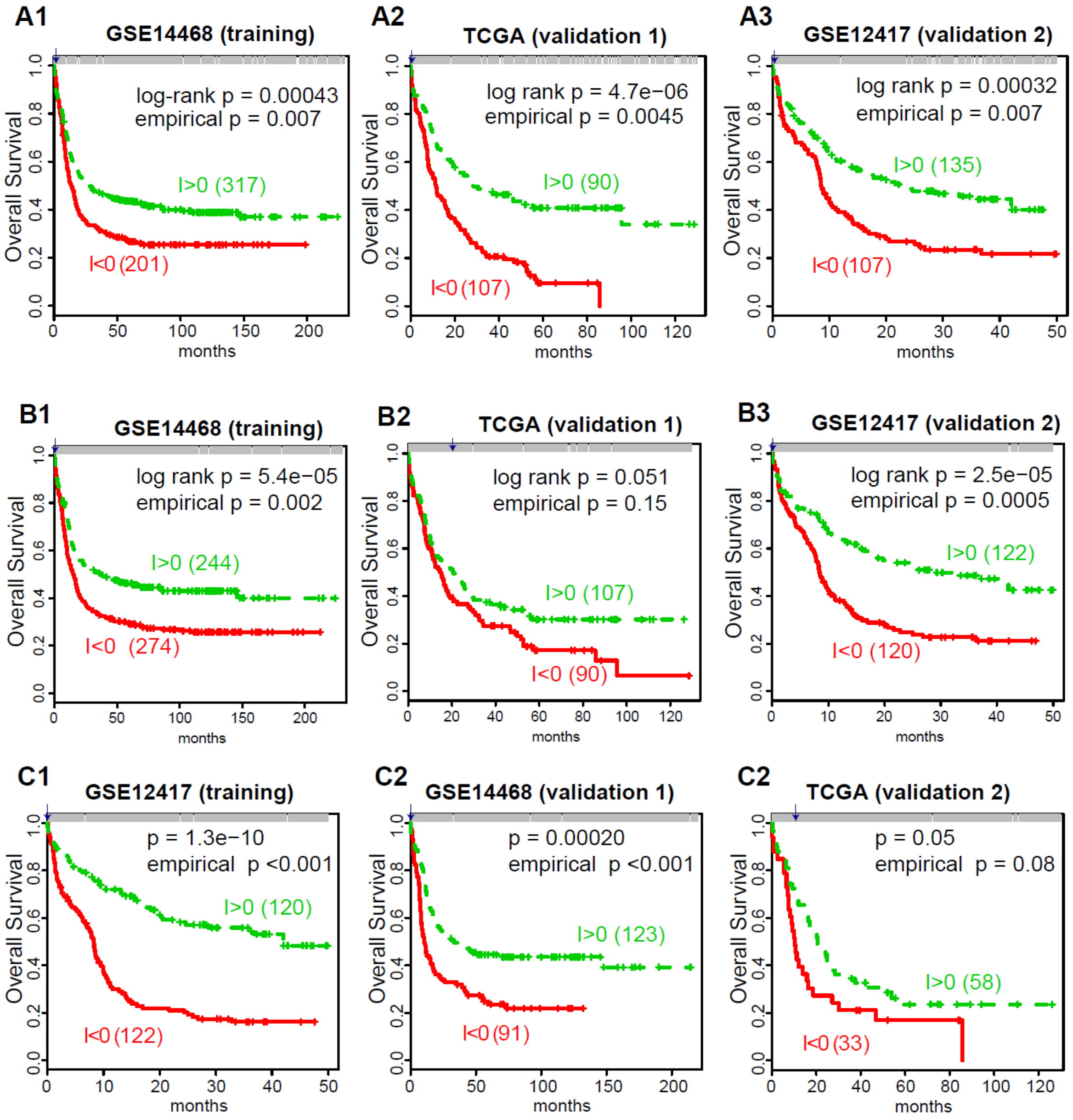

Figure S5. **Kaplan–Meier plots of patients with primary AML, based on DNM identified gene-set pairs.** The Relative Effect Analysis with Functional gene-set-Group Pairs (REA-FGP) yielded a prognostic indicator. **Panels A and B** are the results of analysis on patients with all types of AML; whereas **Panel C** is the results of analysis on cytogenetically normal AML patients. **Panel A** compares three LSC- representative gene-sets (30 genes) with four normal control gene-sets (242 genes in Table S2); **Panels B and C** compare the LSC+ representative gene-sets (25 genes) with four normal control gene-sets (359 and 99 genes in Table S3).

Subpanels 1 are the training results. Subpanels 2-3 show the validations. In each sub-panel, top rugs mark the simulated p-values from which we estimated the empirical p-value for the actually observed log-rank p-value, the vertical blue arrow. An indicator *I* of less than 1 significantly indicates worse prognosis.

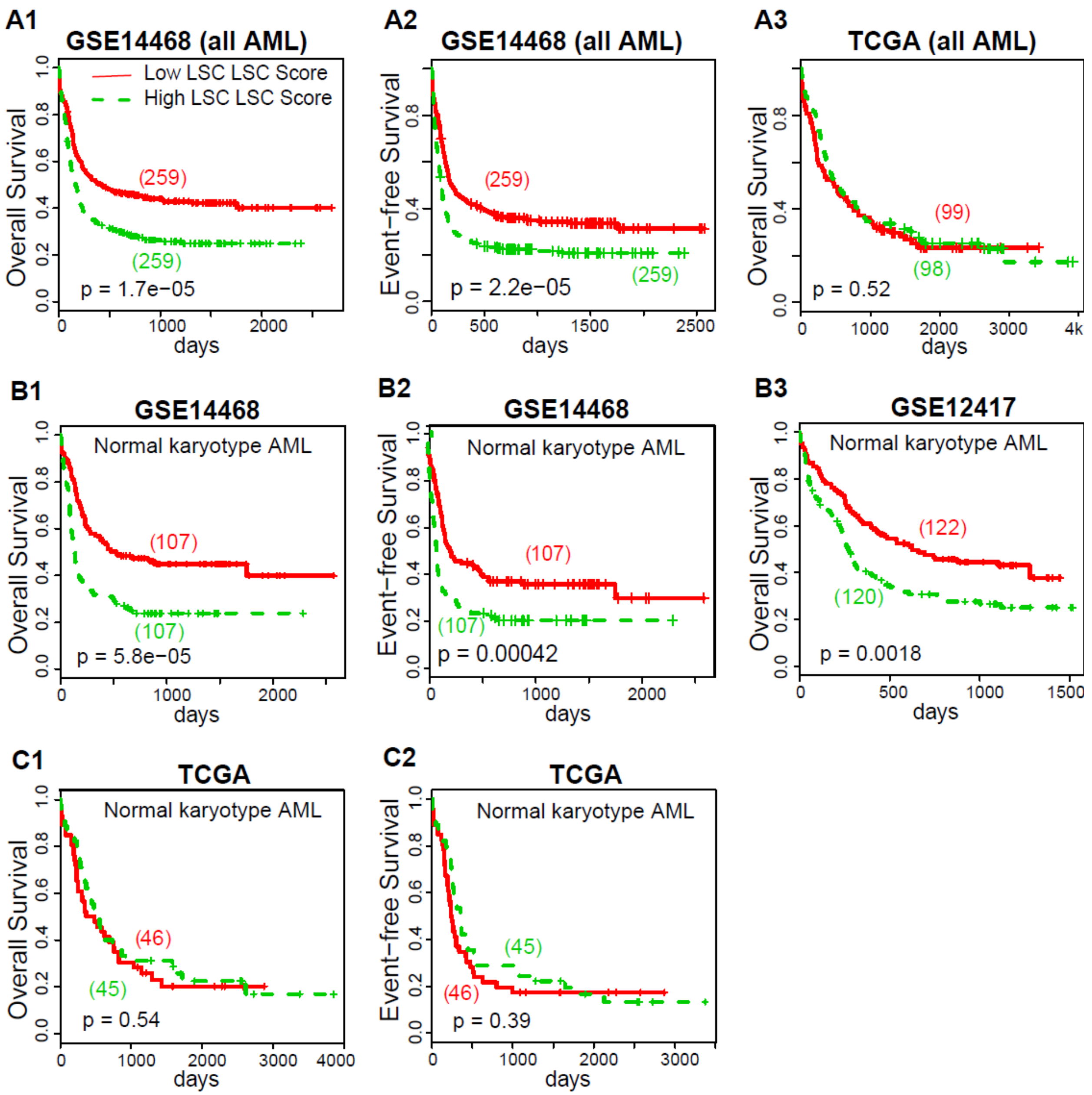

Figure S6. **Kaplan–Meier plots of patients with primary AML, based on Gentles "LSC signature" (Gentles AJ, et al. JAMA 2010, 304(24):2706).** As defined by Gentles et al, the sum of weighted 31 genes gives a LSC score for each patient, and the median of scores splits patients into two groups. **Panel A** are the results of analysis on patients with all types of AML; whereas **Panels B and C** are the results of analysis on patients with normal karyotype AML. In each sub-panel, red solid lines indicate the survival of patients with low LSC scores while the green dashed lines indicate the survive of patients with high LSC scores. The Gentles weightings determined with CD34 were used here, and the weightings without CD34 generated similar results. We mapped probes from different platforms using the Bioconductor database *biomaRt* v2.22.0 for GSE12417. For the TCGA data, we directly analyzed its level 3 expression data with annotated gene symbols (genome.wustl.edu_LAML.HG-U133_Plus_2.Level_3.1.3.0).
