# Supplementary material for: Systematic computation with functional gene-sets among leukemic and hematopoietic stem cells reveals a favorable prognostic signature for acute myeloid leukemia
Source: BMC Bioinformatics. 2015 Mar 24;16:97. doi: 10.1186/s12859-015-0510-7 (PMC4376348; doi:10.1186/s12859-015-0510-7)
Supplement: Additional file 2: — Text S1. Supplementary results, methods, and the limitation. [file 12859_2015_510_MOESM2_ESM.pdf]

1. Supplementary Results

1.1) Simulation study to find the required sample size and the optimized the parameter *α*

***Theoretical Simulation* –** We performed two simulation studies to evaluate gene-set analysis methods in the context of statistical power and type-I error, similar to a prior study [1]. The purpose of the first study was to find a minimal sample size to guarantee statistical power for gene-set signature identification, whereas the second was to decide the optimal parameter α for the FAIME algorithm and reveal the effect of gene-set size when detecting gene-set signatures. We used four different simulation scenarios for each study, considering strong and weak signal-to-noise ratios (1 and 0.5 respectively) and altered fractions of differentially expressed (DE) genes (50% and 80% respectively).

In the first simulation study, we created mimic datasets with increased sample sizes and two gene-sets (GSs) each of a fixed GS size (30 out of 5000 genes), where one GS was differentially expressed and the other was not. Larger sample size noticeably increased statistical power but not type-I error. Using 20 samples per group, we could identify GS signatures in strong signal-to-noise-ratio scenarios (**Fig. S1-C3, D3,** 60-95% statistical power), using either FAIME.α, GSEA, or GSVA. Additionally, all methods provided effective control of the type-I error rate when GS having more than 30 gene members. GSEA and GSVA slightly outperformed FAIME.1 in scenarios with weak signal-to-noise-ratio (**Fig. S1-A, B**), while all three algorithms performed equally in the scenarios with strong signal-to-noise-ratio (**Fig. S1-C, D**). We used the Bioconductor packages PGSEA and GSVA to apply the GSEA and GSVA algorithms to all gene-sets with five or more genes from the MSigDB.

In the second simulation study, we repeated all of the aforementioned steps but tested on simulated GSs of different gene sizes (*x*=10, 20, 80, and 100). For small GSs (10-20 genes), FAIME showed statistical advantages with consistently low type-I error (**Fig. S1- a1, b1, b2**). For other GSs (>=30 genes), the larger a GS size the lower a statistical power derived from the same sample size in all simulation scenarios, suggests that 40-60 samples per group are required to test larger GS (>100 genes).

Alternatively, applying a larger parameter α to FAIME will benefit the analysis of small GS via weighting more sharply towards the leading expression ranks (**Equation 1**). In both simulation studies, FAIME with *α* =1 by default worked more accurately than *α* =5 or 10, whereas the latter effectively controlled the type-I error for small GSs.

1.2) Summary of published LSC-associated gene signatures

Table S1 summarizes nine multiple-gene studies pertaining to LSCs, three of which the original authors checked prognosis in primary AML samples. These gene signatures can be divided into the three categories below. For the comparison between AML LSC+ and normal HSC+, we excluded those “HSC” samples from **Table S1** with detectable expression of only CD34+ (neither the mature blood lineages nor

their committed progenitors markers) because normal CD34+ mononuclear cells contain hematogones (B lymphocyte precursors) and CD34+ megakaryocytes.

Stemness - We obtained an union set of three published multi-gene signatures, which we refer to as "LSCvLPC" (**Fig. S2)**. Three studies respectively identified a gene-list that significantly distinguishes LSC+ populations ($CD34^{+}CD38^{-}$) from more mature fraction of the leukemia clone, the leukemia progenitor cell (LPC) populations ($CD34^{+}CD38^{+}$), purified from the same AML samples [2-4]. Ishikawa *et al* demonstrated that such LSC+ exclusively recapitulates AML and retains self-renewal capacity *in vivo* [2]. Gentles *et al* showed that expression of their gene-signature in bulk primary AML tumor samples was associated with clinical outcomes in four independent patient cohorts (n = 1047) [4]. Unfortunately, few overlaps exist among these three signatures. Only the cell surface marker CD38 was expressed lower in LSC than in LPC (**Fig. S2A1**). Additionally, a potential non-stem-cell signature was involved, as both studies ignored the fact that the $CD34^{+}CD38^{+}$ subpopulation may also resides LSCs [5-8]. However, these three studies are more biologically sensitive by comparing pairwise cell sub-populations purified from the same patients. Therefore by joining these three published gene signatures, we could generally characterize leukemia stemness.

An improved study by Eppert *et al* verified four divergent LSC+ fractions using xenograft models [5]. They identified multiple LSC+ fractions in AML samples using a sensitive xenograft assay and then identified a LSC-specific signature more highly expressed in LSC+ than in LSC-. They showed this signature to be a significant and independent predictor of patient survival (n=445). They also identified a signature specific to normal HSC+ fractions but not normal mature fractions and showed its prognosis in primary AML [5].

Malignancy - We joined two published gene signatures which we refer to as "LSCvNHSC". Comparing refined LSC+ populations ($Lin^{-}CD34^{+}CD38^{-}CD90^{-}$) with normal hematopoietic stem cell enriched (HSC+) populations ($Lin^{-}CD34^{+}CD38^{-}CD90^{+}$), Majeti *et al* identified a LSC specific signature [9]. Similarly, Saito *et al* identified genes with significantly higher expression in AML LSC+ ($CD34^{+}CD38^{-}$) than in normal HSC+ ($CD34^{+}CD38^{-}$) [10].

Other dataset - De Jonge *et al* reported 50 genes that specifically high-expressed in AML $CD34^{+}$ but not AML $CD34^{-}$ fractions when compared with normal $CD34^{+}$ compartment [11]. Based on the summed expression level of three out of the 50 genes, they suggested that a high transcript level of CD34+ cells was associated with significant unfavorable overall survivals in two independent cohorts (n=381) of normal karyotype AML. However, the statistical significance is mild as it can be achieved after trichotomizing bulk samples rather than dichotomizing.

2. Supplementary Methods

2.1) Data process

Gene expression data. We downloaded the normalized gene expression values and transformed the values to a logarithmic scale (log2) when required. One RNA-seq dataset of normal HSC samples was provided by the authors [12]. The measurements were scaled in Reads Per Kilobase of exon model per Million mapped reads (RPKM) format [13], and genes were annotated by Ensemble IDs.

MSigDB employs only the official gene symbols and Entrez IDs, leading us to use the Bioconductor package *biomaRt* (version 2.16.0) to map all probes on a microarray to Entrez gene IDs as well as all Ensemble IDs of RNAseq to official gene symbols. To get the best coverage for custom microarray platforms, we also incorporated the corresponding custom annotation files downloaded from GEO.

Functional gene-sets - We downloaded three categories of previously defined gene-sets from Molecular Signature Database (MSigDB, version 4.0) [14]: canonical representations of biological processes compiled by domain experts (from BIOCARTA, KEGG, and REACTOME) (N=1320), gene-sets representing expression signatures of genetic and chemical perturbations (CGP, N=3402), and transcription factor or microRNA targets based on conserved cis-regulatory motifs from a comparative analysis of the human, mouse, rat, and dog genomes (N=836). The average sizes of gene-members in these three gene-set categories vary from 29 to 233. CGP gene-sets have on average 45 gene-members per set (range of 5 to 1972).

2.2) Hypergeometric probability analysis

To estimate the probability of observing n=6 overlapping genes among three instances of random sampling (**Table S2**, three DNM GSs using FAIME.5 profiles), we did the following modeling: out of a space of N=22000 genes, we randomly picked a group of A=11 genes, recorded them and put them back, then repeated for a group of B=6 genes and C=19 genes. We wished to compute the probability that out of the 36=A+B+C recorded genes, exactly n=6 genes appear twice and exactly A+B+C-2n=24 genes appear once.

Note that for a gene to appear exactly twice, it must appear in two of the three groups and not in the third. In particular, any number 0 ≤ n-k ≤ n of the repeated genes may have one of its groups be C, which means the remaining k genes must be in both A and B. We can arbitrarily pick A, and given those 11 distinct genes, there are a total of $\binom{N}{B}\binom{N}{C}$ ways to pick B and C. We can pick the k genes from A that will be repeated in B in $\binom{A}{k}$ ways, and we can pick the remaining B-k genes in B in $\binom{N-A}{B-k}$ ways.

There are a total of A+B-k genes in the union of A and B, *A+B-2k=17-2k* of which only appeared once. Since we didn't observe any gene appearing in all three of the groups but we needed to have *n-k* more genes repeated for a total of *n* repeated genes, we chose the *n-k* genes from the non-repeated pool of *17-2k* genes in A and B to also appear in C. This could be done in $\binom{A+B-2k}{n-k}$ ways, and we could pick the remaining *C-(n-k)=13+k* genes in C in $\binom{N-(A+B-k)}{C-(n-k)}$ ways.

Summing over all k's, we have our desired probability that out of the *(A+B+C)=36* recorded genes, exactly *(A+B+C-2n)=24* genes appear once and exactly *n=6* genes appear twice is:

$$p(x=n \mid A,B,C,N) = \sum_{k=0}^{n} \frac{\binom{A}{k}\binom{N-A}{B-k}}{\binom{N}{B}} \frac{\binom{A+B-2k}{n-k}\binom{N-A-B+k}{C-n+k}}{\binom{N}{C}}.$$

2.3) Gene Ontology sematic similarity evaluation

The Gene Ontology (GO) sematic similarity between pair-wise gene members of the identified 25- or 30-gene signature was estimated using Lin's method [15]. Given two genes *x* and *y*, Lin's method assigns a sematic similarity score, $Sim(x,y) = 2\frac{sim(x,y)}{sim(x,x)+sim(y,y)}$. We employed the Bioconductor package *GOSim* to run the calculation (similarity="funSimMax", similarityTerm="relevance", normalization=TRUE) [16], respecting the GO biological process and GO molecular function respectively. To estimate the empirical p-value of an observed similarity score, we ran the same calculation for 1000 pairs of randomly selected genes.

2.4) Enrichment analysis (EA) on the published gene lists

For LSC stemness (LSC+ compared to LSC-), we focused on the gene-sets that were identified by two out of the three gene lists: the joint gene-set LSCvLPC, AML stemness, and normal stemness in Eppert study (**Fig. S2A2**, yellow circles). For LSC malignancy (AML LSC+ compared to normal HSC+), we focused on the gene-sets identified by both the jointed LSCvNHSC list (**Fig. S2A2**, dash-lined pink circles) and LSC highly expressed gene-sets in De Jonge's study (**Fig. S2A2**, blue circles). To interrogate the LSC stemness, we reported the gene-sets that were significantly enriched in two out of the three gene-lists (**Fig. S2A2**, yellow circles). Specifically for enriched gene-set in Eppert study (GES30377), we merged our EA identification with three additional author-reported LSC-associated gene-sets (BENPORATH PRC2 TARGETS, PARK HSC VS MULTIPOTENT PROGENITORS UP, and IVANOVA HEMATOPOIESIS EARLY PROGENITOR). We did so because these three gene-sets are LSC-specific

(LSC $p<0.05$ but HSC $p>0.05$, see the Table S14 in the original publication) and can be mapped into the newest MsigDB v4.1 version. To interrogate the malignancy, we reported the gene-sets that were significantly enriched in both two gene-lists (**Fig. S2A2**, blue and pink circles). Note that there are no overlaps between stemness and malignancy for the LSC+ population, suggesting their distinct properties.

As we observed before [17], functional-level EA exhibits remarkably greater cross-study reproducibility than gene-level significance analysis. We observed 60% repetition of enriched canonical pathways on average (5-100%, FET $p<0.05$, gene count≥3, **Fig. S2B**) and 56% repetition of targets of chemical and genetic perturbations (15%-73%).

3. Limitation

Our study has some limitations. First, FAIME is designed to identify up-regulated or down-regulated gene-sets. However, genes in the same pathway are not always differentially expressed in the same direction. Some disease condition associated pathways may contain both up- and down-expressed genes caused by feedback loops, such as the p53 pathway [18]. Whereas GSVA and GSAA [19] can identify this type of concerted gene-set expression using a non-parametric KS-test. Second, based on an arbitrary cutoff of significance at the gene-set level, identifications that have borderline differential activities or modest effect size may be lost when applying inter-group comparison for both FAIME and GSVA methods. On the other hand, some false positive identifications met the criteria of significance, suggesting that we should apply FAIME/GSEA on the data with symmetric differential expression background at gene level. Third, the 'inter-dataset' normalization is a straightforward use of Z-scores. This type of standardization has been successfully applied to integrate gene-set scores of differentially expressed genes and of trait-associated genetic markers [19]. Even so, a standard deviation parameter for the normalization of all gene-sets, including over- and under-represented gene-sets, may reduce the over-representation of some gene-sets while increasing the under-representation of others. In such cases, distinct standard deviation parameters for the over- and under-represented gene-sets are suggested for future discussion. Finally, gene-sets only reflect an approximation of biological functions or pathways and are pre-defined. Only a subset of genes within a set may contribute to a gene-set expression signature. Different gene-sets may have similar signatures pertaining to the same phenotype, owing to either an overlap between the gene-sets or co-regulation of non-overlapping gene-sets. Grouping correlated gene-sets and extending the interaction of their gene members, perhaps by modeling on additional information, is a potentially promising approach to define new functional gene-sets.

Reference for Supplementary Material:

1. Hanzelmann S, Castelo R, Guinney J: **GSVA: gene set variation analysis for microarray and RNA-seq data**. *BMC Bioinformatics* 2013, **14**:7.
2. Ishikawa F, Yoshida S, Saito Y, Hijikata A, Kitamura H, Tanaka S, Nakamura R, Tanaka T, Tomiyama H, Saito N *et al*: **Chemotherapy-resistant human AML stem cells home to and engraft within the bone-marrow endosteal region**. *Nat Biotechnol* 2007, **25**(11):1315-1321.

3. Gal H, Amariglio N, Trakhtenbrot L, Jacob-Hirsh J, Margalit O, Avigdor A, Nagler A, Tavor S, Ein-Dor L, Lapidot T *et al*: **Gene expression profiles of AML derived stem cells; similarity to hematopoietic stem cells**. *Leukemia : official journal of the Leukemia Society of America, Leukemia Research Fund, UK* 2006, **20**(12):2147-2154.
4. Gentles AJ, Plevritis SK, Majeti R, Alizadeh AA: **Association of a leukemic stem cell gene expression signature with clinical outcomes in acute myeloid leukemia**. *JAMA* 2010, **304**(24):2706-2715.
5. Eppert K, Takenaka K, Lechman ER, Waldron L, Nilsson B, van Galen P, Metzeler KH, Poeppl A, Ling V, Beyene J *et al*: **Stem cell gene expression programs influence clinical outcome in human leukemia**. *Nat Med* 2011, **17**(9):1086-1093.
6. Taussig DC, Miraki-Moud F, Anjos-Afonso F, Pearce DJ, Allen K, Ridler C, Lillington D, Oakervee H, Cavenagh J, Agrawal SG *et al*: **Anti-CD38 antibody-mediated clearance of human repopulating cells masks the heterogeneity of leukemia-initiating cells**. *Blood* 2008, **112**(3):568-575.
7. Yoshimoto G, Miyamoto T, Jabbarzadeh-Tabrizi S, Iino T, Rocnik JL, Kikushige Y, Mori Y, Shima T, Iwasaki H, Takenaka K *et al*: **FLT3-ITD up-regulates MCL-1 to promote survival of stem cells in acute myeloid leukemia via FLT3-ITD-specific STAT5 activation**. *Blood* 2009, **114**(24):5034-5043.
8. Goardon N, Marchi E, Atzberger A, Quek L, Schuh A, Soneji S, Woll P, Mead A, Alford KA, Rout R *et al*: **Coexistence of LMPP-like and GMP-like leukemia stem cells in acute myeloid leukemia**. *Cancer Cell* 2011, **19**(1):138-152.
9. Majeti R, Becker MW, Tian Q, Lee TL, Yan X, Liu R, Chiang JH, Hood L, Clarke MF, Weissman IL: **Dysregulated gene expression networks in human acute myelogenous leukemia stem cells**. *Proc Natl Acad Sci U S A* 2009, **106**(9):3396-3401.
10. Saito Y, Kitamura H, Hijikata A, Tomizawa-Murasawa M, Tanaka S, Takagi S, Uchida N, Suzuki N, Sone A, Najima Y *et al*: **Identification of therapeutic targets for quiescent, chemotherapy-resistant human leukemia stem cells**. *Science translational medicine* 2010, **2**(17):17ra19.
11. de Jonge HJ, Woolthuis CM, Vos AZ, Mulder A, van den Berg E, Kluin PM, van der Weide K, de Bont ES, Huls G, Vellenga E *et al*: **Gene expression profiling in the leukemic stem cell-enriched CD34+ fraction identifies target genes that predict prognosis in normal karyotype AML**. *Leukemia : official journal of the Leukemia Society of America, Leukemia Research Fund, UK* 2011, **25**(12):1825-1833.
12. Hu G, Schones DE, Cui K, Ybarra R, Northrup D, Tang Q, Gattinoni L, Restifo NP, Huang S, Zhao K: **Regulation of nucleosome landscape and transcription factor targeting at tissue-specific enhancers by BRG1**. *Genome Res* 2011, **21**(10):1650-1658.
13. Mortazavi A, Williams BA, McCue K, Schaeffer L, Wold B: **Mapping and quantifying mammalian transcriptomes by RNA-Seq**. *Nat Methods* 2008, **5**(7):621-628.
14. Liberzon A, Subramanian A, Pinchback R, Thorvaldsdottir H, Tamayo P, Mesirov JP: **Molecular signatures database (MSigDB) 3.0**. *Bioinformatics* 2011, **27**(12):1739-1740.
15. Lin D: **An Information-Theoretic Definition of Similarity**. In: *Proceedings of the Fifteenth International Conference on Machine Learning.* Morgan Kaufmann Publishers Inc. San Francisco, CA, USA 1998: 296-304.
16. Frohlich H, Speer N, Poustka A, Beissbarth T: **GOSim--an R-package for computation of information theoretic GO similarities between terms and gene products**. *BMC Bioinformatics* 2007, **8**:166.
17. Yang X, Regan K, Huang Y, Zhang Q, Li J, Seiwert TY, Cohen EE, Xing HR, Lussier YA: **Single sample expression-anchored mechanisms predict survival in head and neck cancer**. *PLoS Comput Biol* 2012, **8**(1):e1002350.

18. Harris SL, Levine AJ: **The p53 pathway: positive and negative feedback loops**. *Oncogene* 2005, **24**(17):2899-2908.
19. Xiong Q, Ancona N, Hauser ER, Mukherjee S, Furey TS: **Integrating genetic and gene expression evidence into genome-wide association analysis of gene sets**. *Genome Res* 2012, **22**(2):386-397.
