# Supplementary material for: Systematic computation with functional gene-sets among leukemic and hematopoietic stem cells reveals a favorable prognostic signature for acute myeloid leukemia
Source: BMC Bioinformatics. 2015 Mar 24;16:97. doi: 10.1186/s12859-015-0510-7 (PMC4376348; doi:10.1186/s12859-015-0510-7)
Supplement: Additional file 3: — Text S2. R Code and demonstration for the function rungene2pathway. [file 12859_2015_510_MOESM3_ESM.pdf]

Vignette: Systematic computation with functional gene-sets among leukemic and hematopoietic stem cells reveals a favorable prognostic signature for acute myeloid leukemia

Bin Wang, Xinan Yang

Thursday, January 08, 2015

This Vignette includes R source codes to run the FAIME.5 algorithm using demo data.

We introduce the function *rungene2pathway* which is an improved tool for the Functionally Analyzing Individualized Microarray and next generation sequencing data (FAIME) by taking into account of previously defined gene-sets. It compares the cumulative quantitative effects of genes inside an ontology (set of functionally related genes) with those outside, thus overcoming a number of difficulties in prior gene-set enrichment methods [1].

R source code

The input of the function rungene2pathway:
- **dat**: A data frame or matrix of gene expression measurements. The rows of dat correspond to genes, and the columns correspond to samples. Note that official gene symbols must label the rows, and the values contained in dat should be either finite or NA.
- **gsmap**: A list or an R GSA.gene-sets object to define gene-set. Users can call the *GSA.read.gmt* function in the R package **GSA** to load customized gene-sets with a *.gmt* format.
- **alpha**: A positive integer, 5 by default. A higher value puts more weights on the most highly-valued ranks than the lower-valued ranks.
- **logCheck**: A Boolean value, FALSE by default. When being TRUE, the function takes the log-transformed values of all values in *dat* when any value is larger than 20.
- **na.rm**: A Boolean value indicates whether to keep missing values when the parameter method="FAIME". By default, it is FALSE.

Below are the R scripts for the function *rungene2pathway* which calls the internal function *FAIME*:

```
rungene2pathway <- function(dat,gsmap,alpha=5,logCheck=FALSE,method=c("FAIME"),na.rm=FALSE){
    if(missing(method)){method="FAIME"}
    if(missing(alpha)){alpha=5}
    if(missing(na.rm)){na.rm=FALSE}
    if(missing(logCheck)){logCheck=FALSE}
    if(is.data.frame(dat)==FALSE & is.matrix(dat)==FALSE){
      stop("Error: input should be a data frame or a matrix")}

    if(class(gsmap)=="GSA.genesets"){
      seeds <- gsmap$geneset.names
      res <- matrix(nrow=length(seeds), ncol=ncol(dat))
      for(i in 1:length(seeds)){
        for(j in 1:ncol(dat)){
          res[i,j] <- FAIME(sampleExp=dat[,j], GeneID=toupper(rownames(dat)),
                            Geneset=toupper(gsmap$genesets[[i]]),
```

```
                              alpha=alpha, logCheck=logCheck,na.rm=na.rm)
        }#j loop
      }#i loop
    }else if(class(gsmap)=="list"){
      if(is.null(names(gsmap))){stop ("please give the names of gsmap as a list")}
      seeds <- names(gsmap)
      res <- matrix(nrow=length(seeds), ncol=ncol(dat))
      for(i in 1:length(seeds)){
        for(j in 1:ncol(dat)){
          res[i,j] <- FAIME(sampleExp=dat[,j],GeneID=toupper(rownames(dat)),
                              Geneset= toupper(gsmap[[i]]), alpha=alpha,
                              logCheck=logCheck,na.rm=na.rm)
        }#j loop
      }#i loop
    }#class loop

    rownames(res) <- seeds
    colnames(res) <- c(paste(colnames(dat),"2pathscore",sep=""))
    print("gene2pathay calculates score....... done")
    return(res)
  }

FAIME <- function(sampleExp, GeneID, Geneset, alpha, logCheck,na.rm){
    if(class(sampleExp)!="numeric"){sampleExp <-as.numeric(levels(sampleExp))[sampleExp]}
    if(logCheck){if(max(sampleExp, na.rm=TRUE) > 20) {sampleExp <- log2(sampleExp)}}
    if(length(sampleExp)!=length(GeneID)){
      stop("Error: GeneID information is missing or not correct!")}

    N <- length(GeneID)
    GeneID_NaInSet <- GeneID[which(!GeneID %in% Geneset)]

    #Step 1: Calculation of weighted rank of gene expression
    rankedExp <- rank(sampleExp, na.last="keep")
    rankscore <- rankedExp*exp((rankedExp/N*alpha)-alpha)

    #Step 2:
    x1 <- which(GeneID %in% Geneset)
    x2 <- which(GeneID %in% GeneID_NaInSet)

    ST <- sum(rankscore[x1], na.rm=TRUE)/length(x1)
    SN <- sum(rankscore[x2], na.rm=TRUE)/length(x2)
    y <- sum(ST, -SN, na.rm=na.rm)

    return(y)
  }
```

Example

Step 1. Get gene expression data "leukemia" from the R package GSVA.

A filtering of probesets with the lowest IQR is performed in the demo to save running time but not necessary.

```
library(GSEABase)
library(GSVAdata)
library(genefilter)
library(GSVA)
data(leukemia)
dim(leukemia_eset)
## Features  Samples
##    12626       37
filtered_eset <- nsFilter(leukemia_eset, require.entrez=TRUE, remove.dupEntrez=TRUE,
                          var.func=IQR, var.filter=TRUE, var.cutoff=0.5, filterByQuantile=TRUE,
                          feature.exclude="^AFFX")
leukemia_filtered_eset <- filtered_eset$eset
dat_exp<-as.data.frame(exprs(leukemia_filtered_eset))
dim(dat_exp)
## [1] 4318   37
head(dat_exp)
##             CL2001011101AA.CEL CL2001011102AA.CEL CL2001011104AA.CEL
## 907_at               11.857516          11.161085          11.512466
## 35430_at             10.328026           9.494069           9.012711
## 36841_at              9.591560           9.820928           9.347952
## 38924_s_at           12.417347          11.706729          12.392675
## 36023_at             11.013613          12.619952          11.175109
## 191_at                9.488507           9.775179           9.439493
##             CL2001011105AA.CEL CL2001011109AA.CEL CL2001011110AA.CEL
## 907_at               10.185201          12.231562          11.918172
## 35430_at             10.282458           8.202723           8.241378
## 36841_at              9.616633          10.134252          10.088614
## 38924_s_at           12.842632          11.245247          11.656117
## 36023_at             11.971174           9.980090           9.438358
## 191_at                9.595062           9.429010           9.625638
##             CL2001011111AA.CEL CL2001011112AA.CEL CL2001011113AA.CEL
## 907_at               11.491475          12.213642          11.793422
## 35430_at              8.255273           9.090225           8.816643
## 36841_at              9.605967           9.503621           9.528071
## 38924_s_at           11.804745          12.108045          11.824156
## 36023_at             10.438486           9.971472          10.924746
## 191_at                9.511813           9.418150           9.424093
##             CL2001011114AA.CEL CL2001011116AA.CEL CL2001011118AA.CEL
## 907_at               11.084444          11.726246          11.798460
## 35430_at              9.897831           8.501331           8.281598
## 36841_at              9.462195           9.559597           9.403876
## 38924_s_at           11.808983          11.275397          11.587586
## 36023_at             11.235509          10.536676          10.410933
## 191_at                9.486785           9.569882           9.882518
##             CL2001011120AA.CEL CL2001011121AA.CEL CL2001011122AA.CEL
## 907_at               12.321334          11.788719          12.197492
## 35430_at              8.511181           9.343495           9.058465
## 36841_at              9.855197           9.523719           9.352326
## 38924_s_at           11.436977          12.212075          11.871556
## 36023_at             10.514632          11.047266          10.608633
## 191_at                9.850949           9.635322           9.731658
##             CL2001011134AA.CEL CL2001011150AA.CEL CL2001011151AA.CEL
## 907_at               11.667078          11.444946          11.689310
```

```
## 35430_at             9.123565          10.020491           9.642977
## 36841_at             9.505989           9.120245           9.470750
## 38924_s_at          12.568188          12.280862          12.007505
## 36023_at            10.818542          10.823133          10.388536
## 191_at               9.499381           9.360173           9.344782
##            CL2001011153AA.CEL CL2001011154AA.CEL CL2001011126AA.CEL
## 907_at              11.636323          10.638600          11.357611
## 35430_at            10.258735           8.813048           8.923003
## 36841_at             9.281449           9.627975           9.858323
## 38924_s_at          12.256351          10.903222          12.076050
## 36023_at            11.514675          10.341163          10.637183
## 191_at               9.592377           9.469548           9.343042
##            CL2001011127AA.CEL CL2001011128AA.CEL CL2001011129AA.CEL
## 907_at              12.006894          11.836165          11.551714
## 35430_at             8.035410           8.278299           9.024235
## 36841_at             9.787795          10.185504           9.715688
## 38924_s_at          12.110634          11.968350          11.399876
## 36023_at            11.538635          10.692843          10.486363
## 191_at               9.356517           9.335555           9.927592
##            CL2001011130AA.CEL CL2001011131AA.CEL CL2001011132AA.CEL
## 907_at              10.741771          12.049258          12.156861
## 35430_at            10.003668           7.965007           7.655641
## 36841_at             9.225892          10.005552           9.934143
## 38924_s_at          12.481346          11.444596          11.304336
## 36023_at            11.170412          10.549629          10.284485
## 191_at               9.711779           9.580291           9.638747
##            CL2001011133AA.CEL CL2001011138AA.CEL CL2001011139AA.CEL
## 907_at              10.402571          11.857256           9.698532
## 35430_at             7.778797           9.111072           8.952164
## 36841_at             9.874878           9.233652           9.307411
## 38924_s_at          11.644874          12.645314          11.646653
## 36023_at            11.141781          11.342956          10.809038
## 191_at               9.849557           9.454219           9.854332
##            CL2001011140AA.CEL CL2001011142AA.CEL CL2001011143AA.CEL
## 907_at              10.663037          11.594571          10.930493
## 35430_at             7.563916           8.061963           8.315588
## 36841_at            10.114030           9.757547           9.870069
## 38924_s_at          10.970311          11.241144          11.443139
## 36023_at            11.968539          10.211034          10.382740
## 191_at               9.946538           9.979112           9.748915
##            CL2001011144AA.CEL CL2001011146AA.CEL CL2001011149AA.CEL
## 907_at              10.284027          11.914410          12.013266
## 35430_at             9.540942           8.591968           8.836134
## 36841_at             9.600789          10.172181           9.457279
## 38924_s_at          12.063623          11.500465          11.598560
## 36023_at            11.461717          10.785368          10.563032
## 191_at               9.997164          10.065955           9.550446
##            CL2001011152AA.CEL
## 907_at              11.246136
## 35430_at             9.414451
## 36841_at             9.218744
## 38924_s_at          12.144874
## 36023_at            10.699175
```

```
## 191_at                9.682976
```

Step 2. Replace Affymetrix probeset ID with gene hgnc_symbols as the row-names of the R data object "leukemia".

As a demo, we run FAIME only with the first 4 samples.
If there are more than one rows of expression for the same gene, we recommend collapsing this gene into one row with the highest value (maximum) within the column for that gene. The Bioconductor package *WGCNA* provides several methods to select the row. In this demo, we use the first row for simplicity.

```
library(biomaRt)
ensembl = useMart("ensembl")
ensembl = useDataset("hsapiens_gene_ensembl",mart=ensembl)
ID_symbol <- getBM(attributes=c("affy_hg_u95a","hgnc_symbol"), filters = "affy_hg_u95a",
                   values = rownames(dat_exp), mart = ensembl)
ID_symbol<-ID_symbol[ID_symbol$hgnc_symbol!="",]
mdat <- as.data.frame(matrix(, nrow = 0, ncol = 2, byrow = TRUE,
               dimnames = list(NULL,c("affy_hg_u95a", "hgnc_symbol"))))
for(i in 1:length(unique(ID_symbol$hgnc_symbol))){
  sub<-ID_symbol[ID_symbol$hgnc_symbol==unique(ID_symbol$hgnc_symbol)[i],]
  mdat<-rbind(mdat,sub[1,])
}
dat_exp$affy_hg_u95a<-rownames(dat_exp)
dat_exp_fil<-merge(dat_exp,mdat,by="affy_hg_u95a",all=F)
rownames(dat_exp_fil)<-dat_exp_fil$hgnc_symbol
dat_exp_fil<-dat_exp_fil[,2:5]
dim(dat_exp_fil)
## [1] 4449    4
head(dat_exp_fil)
##       CL2001011101AA.CEL CL2001011102AA.CEL CL2001011104AA.CEL
## MAPK3          11.354426          10.932543          11.185906
## TIE1            9.185470           8.823661           8.687186
## DUSP1          13.717107          14.469777          13.713944
## HINT1          12.683549          13.818987          13.233068
## DYRK4           9.205476          10.190301           9.117263
## YWHAE          10.323587           9.434503          11.261651
##       CL2001011105AA.CEL
## MAPK3          11.251631
## TIE1            8.958305
## DUSP1          13.415991
## HINT1          12.915500
## DYRK4           9.688376
## YWHAE           9.609229
```

Step 3. Download the genesets data (*Msigdb* version 4, collection *c2.cgp*) from the website to local directory.

The users can find the MSigdb collection at: http://www.broadinstitute.org/gsea/msigdb/collections.jsp
Please assign the parameter *mygmt* to your local directory before running the demo.

```r
library(GSA)
mygmt<-"G:/projects/Share/MsigDB/c2.cgp.v4.0.symbols.gmt"
Msig<-GSA.read.gmt(mygmt)
```

Step 4. Run the function with the default parameter set.

```r
res<-rungene2pathway(dat=dat_exp_fil,gsmap=Msig)
## [1] "gene2pathay calculates score....... done"
head(res)
##                                       CL2001011101AA.CEL2pathscore
## NAKAMURA_CANCER_MICROENVIRONMENT_UP                     -578.18675
## NAKAMURA_CANCER_MICROENVIRONMENT_DN                     -408.05464
## WEST_ADRENOCORTICAL_TUMOR_MARKERS_UP                     -91.37237
## WEST_ADRENOCORTICAL_TUMOR_MARKERS_DN                     139.48609
## WINTER_HYPOXIA_UP                                        380.22178
## WINTER_HYPOXIA_DN                                         66.89387
##                                       CL2001011102AA.CEL2pathscore
## NAKAMURA_CANCER_MICROENVIRONMENT_UP                     -469.66161
## NAKAMURA_CANCER_MICROENVIRONMENT_DN                     -384.09149
## WEST_ADRENOCORTICAL_TUMOR_MARKERS_UP                     -32.41900
## WEST_ADRENOCORTICAL_TUMOR_MARKERS_DN                     182.15191
## WINTER_HYPOXIA_UP                                        529.62249
## WINTER_HYPOXIA_DN                                        -24.69069
##                                       CL2001011104AA.CEL2pathscore
## NAKAMURA_CANCER_MICROENVIRONMENT_UP                     -643.64627
## NAKAMURA_CANCER_MICROENVIRONMENT_DN                     -295.01335
## WEST_ADRENOCORTICAL_TUMOR_MARKERS_UP                    -132.99217
## WEST_ADRENOCORTICAL_TUMOR_MARKERS_DN                     223.50142
## WINTER_HYPOXIA_UP                                        538.36660
## WINTER_HYPOXIA_DN                                         21.03335
##                                       CL2001011105AA.CEL2pathscore
## NAKAMURA_CANCER_MICROENVIRONMENT_UP                     -450.80775
## NAKAMURA_CANCER_MICROENVIRONMENT_DN                     -427.75050
## WEST_ADRENOCORTICAL_TUMOR_MARKERS_UP                      35.13746
## WEST_ADRENOCORTICAL_TUMOR_MARKERS_DN                     349.43553
## WINTER_HYPOXIA_UP                                        322.19420
## WINTER_HYPOXIA_DN                                         18.64051
```

Reference:

1. Yang X, Regan K, Huang Y, Zhang Q, Li J, Seiwert TY, Cohen EE, Xing HR, Lussier YA: Single sample expression-anchored mechanisms predict survival in head and neck cancer. PLoS Comput Biol 2012, 8(1):e1002350.
