# Supplementary material for: Systematic computation with functional gene-sets among leukemic and hematopoietic stem cells reveals a favorable prognostic signature for acute myeloid leukemia
Source: BMC Bioinformatics. 2015 Mar 24;16:97. doi: 10.1186/s12859-015-0510-7 (PMC4376348; doi:10.1186/s12859-015-0510-7)
Supplement: Additional file 4: Table S1. — Nine studies and eight published gene lists pertaining to AML LSC. Table S2. LSC- representative DNM gene-sets (30-gene) and their control pairs in the normal state, derived from gene-set profiles using the FAIME.5 algorithm. Table S3. LSC+ representative DNM gene-sets (25-gene) and their control pairs in the normal states, derived from gene-set profiles using the GSVA algorithm. Table S4. Three independent primary AML studies. Table S5. Univariate and multivariate analyses of overall survival in patients with all types of AML, for the LSC+ representative DNM gene-sets. Table S6. The somatic mutations (of genes in the identified DNM gene-sets) and their patient information in the TCGA database. [file 12859_2015_510_MOESM4_ESM.pdf]

Table S1. Nine studies and eight published gene lists pertaining to AML LSC derived from a literature review.

First author	Gal	Ishikawa F	Gentles AJ	De Jonge HJ	Eppert K		Majeti R	Saito Y	Goardon N	Hu
raw data	weizmann.ac.il	CBX21	GSE24006	GSE30029	GSE30377 (GSE30375, 30376)		GSE17054	Not available	E-TABM-978	GSE26501[#]
Mouse model	N/A	(NOD/SCID/IL)2r gamma(null) (NSG)	N/A	N/A	NOD/ShiLtSz-SCID (NOD-SCID)		N/A	NOD-SCID	NOD-SCID or NGS	N/A
Journal	Leukemia	Nat Biotech	JAMA	leukemia	Nature Med		PNAS	SciTransl Med	Cancer Cell	Genome Res
Year	2006	2007	2010	2011	2011		2009	2010	2011	2011
PMID	17039238	17952057	21177505	21760593	21873988		19218430	20371479	21251617	21795385
Last author	Givol D	Shultz LD	Alizadeh AA	Schuringa JJ	Dick JE		Weissman IL	Ishikawa F	Vyas P	Zhao
published LSC induced gene signature (The original table) that we did enrichment analysis	133*	14	31** (Table S2)	50 (Table S5)	42 (48 proes, Table S7)	130^ (147 probes,Table S8)	1702 (Table S3)	259	N/A	N/A
two-group comparision for the published gene-lists$	AML stemness	AML stemness	AML stemness [re-using CBX21)	specific	AML stemness	normal stemness	malignancy	malignancy	N/A	N/A
Validate prognosis in bulk pts	No	No	Yes	Yes	Yes		No	No	No	N/A
platform	HG_U133A	HG-U133_Plus_2	HG-U133_Plus_2 (GPL10881)	Illumina HumanHT-12 V3.0 (GPL6947)	HG_U133A +B	HG_U133A, U133B	HG-U133_Plus_2	HG_U133_Plus_2, Gene 1.0ST	Illumina HumanHT-12 v3.0 (GPL6947)	Illumina Genome Analyzer II

#: RPKM was provided by the authors, sample GSM651554 (SRX037948) in GSE26501 is used.

*: Official gene symbols were downloaded from MsigDB v4.0 under the genesets termed "GAL_LEUKEMIC_STEM_CELL_UP" and "GAL_LEUKEMIC_STEM_CELL_DN".

**: The author reported a signature of 32 highly-and 20 lowly-expressed genes. The gene UBE2T, included in Figure 1, was excluded in the original publication (Table S2) and thus was not analyzed.

^: Re-annotated for the 147 probe-sets using Bioconductor package biomaRt version 2.16.0,

d: Published by Li et al in 2011 to select an optimal microarray probe-set to represent a gene on each corresponding platform.

$: Malignancy: AML LSCs versus normal HSCs; AML stemness: LSC-enriched to LSC-depleted populations in AMLs; normal stemness: normal HSCs versus normal mature cells; specific: CD34+ AML vs CD34+ normal but not CD34- AML vs CD34+ normal

Table S2. LSC- representative DNM gene-sets (30-gene) and their control pairs in the normal state, derived from gene-set profiles using the FAIME.5 algorithm.

ID*	Description of gene-set-cluster	size	Genes in gene-set (PubMed ID)
e	Genes down-regulated in LSC cells compared to LPC cells from AML tumor samples (1)	19	*ANLN, DLGAP5, CD38, ZWINT, IL36B, MS4A3, RNASE2, MND1, CLC, STAR, OLFM4, CCNA1, CCL5, RNASE3, KIAA0101, CSTA, SKA3, DDX53, CPA3*
f	Candidate substrate proteins of AURKA (2)	6	*MBD3, CDC25B, LATS2, DLGAP5, AURKA, CENPA*
g	Genes down-regulated in quiescent vs dividing CD34+ cells isolated from peripheral blood of CML patients (3)	11	*SPC25, CPA3, NDC80, HGF, CSTA, CDK1, MS4A3, MPO, RNASE2, CLC, TOP2A*
Con. (CN, all)	Genes up-regulated in quiescent CD34+ cells isolated from peripheral blood of CML (chronic myeloid leukemia) patients compared to the dividing cells from normal donors. (3)	95	*TPBG, ALOX5, EMP1, TFPI, TSPAN6, ENPP4, LIMCH1, SH3BP5, SORL1, ADAM28, SPTBN1, CCNG2, RBPMS, CXCL2, CD59, CREM, HLX, OPTN, NEAT1, RABGAP1, AKR1C3, TM4SF1, CD40, GLIPR1, CXCL13, ANXA5, GUCY1A3, TNFSF4, CXCL3, IL1B, CH25H, IL8, CD44, TNFSF10, VNN1, BAALC, C3orf14, CTSO, NFAT5, HCP5, SOD2, TCEAL2, TCF7L2, HLA-DPA1, HIST1H2AC, DUSP6, PPFIBP1, HLF, FLJ10038, PTPRC, INHBA, MPZL2, SVIL, EVI2A, DMD, TGFB1I1, ERG, KIAA0125, TRIM22, CRHBP, MLLT3, HLA-DQA1, IFI16, AREG, HLA-DQB1, HLA-DRB4, CD200, CXCL11, TRA2A, MYO5C, CXCL1, PTGER4, BCL11A, SELL, H1F0, IDS, GBP2, CXCL6, TSPAN31, MIR22HG, CLEC2B, PCDH9, TCF4, CYTIP, NRIP1, PMCH, IL18R1, FNBP1, ADAM8, ARMCX2, BIRC3, HIST2H2BE, GPR126, RPL31, CCL19*
Con. (CN, all)	Genes up-regulated in HSC (hematopoietic stem cells) compared to HPC (hematopoietic progenitor cells). (4)	71	*MLLT3, HIST2H2AA3, LIMCH1, PLS3, HIST2H2BE, TFPI, HLA-DQB1, CYBRD1, HSPB1, KLF2, ROBO4, CD37, RBPMS, HIST1H1C, FAM30A, HIST1H2BK, GIMAP6, NPR3, RAPGEF2, IL6ST, KIAA0125, NRIP1, BST2, H1F0, TNFSF10, IDI1, HLA-DQA1, HIST1H2AE, HIST1H2BG, PBXIP1, SPINK2, IDS, INPP4B, COX6B1, SEC62, TRIM8, PGM5, TIPARP, MMRN1, HIST1H3H, HIST1H3D, KLF4, GATA3, GBP2, FAM198B, CLEC2B, HOPX, SPTBN1, ATP8B4, FOSB, HIST1H2BF, GUCY1A3, CD52, LOC404266, PDGFD, BIRC3, MECOM, LOC283070, CRHBP, ARG2, HLF, GUCY1B3, HLA-E, HIST1H2BD, MPL, LOC144481, PRKCH, HOXB3, HIST1H2BC, PPM1F, CEBPB*
Con. (CN, all)	Genes up-regulated in LSC cells compared to LPC (leukemia progenitor) cells from AML tumors. (1)	29	*PCDHGA10, HLF, CD34, LTB, SETBP1, FAIM3, RBPMS, HECA, EVI2A, TMEM200A, LGALSL, GIMAP7, SLC38A1, GIMAP6, MEF2C, SH3BP5, ABCC2, SLC37A3, GIMAP2, HOPX, PION, CCDC48, VNN1, ICAM1, GUCY1A3, MMRN1, FBXO21, BIRC3, EBF3*
Con. (all)	Genes up-rgulated in CD34+ hematopoetic cells by expression of NUP98-HOXA9 fusion off a retroviral vector at 6h. (5)	85	*STK4, IGJ, SLC25A36, SCN2A, CALCRL, HEATR5A, GPR21, GAS2L3, DHRS9, PCDH9, TYRP1, MCTP1, SHISA2, FAM198B, GLIPR1, PLA2G4A, LOC645591, KMO, HOXA4, HPGD, SCML1, GNAI1, FIGN, C3orf80, FKBP5, LOC404266, IL7R, MS4A4A, SERPINI1, XCL1, TEX15, NME7, CD69, ST6GALNAC1, MECOM, DMXL2, SLCO4C1, EDN1, MMP7, ADRBK2, HOXC6, HOXA3, HOXA9, CEP70, OLFM3, PCDH17, KLF5, PLCL1, IL18, INPP4B, TOX, HOXA5, PCOLCE2, PDE4DIP, CMAHP, PTPN13, SPRED1, SEMA3C, GLYATL2, LPAR6, SDPR, CA8, MSX1, NRG4, ZNF503,*

			EVI2A, DACH1, TSPAN12, HOXB3, C5orf30, PBX3, FRMD6, P2RY12, PLN, STAT4, DLX2, CDH9, LIMCH1, USP32, SYBU, CRIM1, S100A10, FLT3, IFNB1, ITGB8

***:** IDs correspond to the node labels in **Fig. 3A**. Con: The control functional gene-set that show significant correlation with any identified gene-set only in normal HSCs (circled nodes in **Fig 3**) and are significantly associated with adverse outcomes in the bulk AML training cohort (log-rank p<0.01, coefficient >0). CN: Control gene-sets were identified by training cytogenetic normal patients. all: Control gene-sets were identified by training all AML patients.

Table S3. **LSC+ representative DNM gene-sets (25-gene) and their control pairs in the normal states, derived from gom gene-set profiles using the GSVA algorithm.**

ID*	Description of gene-set-cluster	size	Genes in gene-sets
a	Amplification hot spot 2: colocolized fragile sites and cancer genes in the 12p13-p11.1 region. (6)	5	*CCND2, ERC1, KRAS, ZNF384, ETV6*
b	Genes down-regulated in LNCaP cells (prostate cancer) treated with forskolin, an activator of PKA pathway. (7)	9	*PIAS1, ATXN3, SESN1, ZBTB10, NAV1, MTERFD2, MAF, SLC30A7, APPBP2*
c	*FOXP3* target genes down-regulated in T lymphocytes after stimulation with *IL2*. (8)	5	*YARS2, GIMAP6, TMIE, STK38, KIF1B*
d	Genes that physically map to the HSC proliferation QTL (quantitative trait locus) Scp2. (9)	6	*LOC728392, GGNBP2, KIF1C, PSMB6, MPO, DYNLL2*
Con. (CN)	Genes up-regulated in colorectal carcinoma samples positive for MSI (microsatellite instability) compared to the MSI negative ones. (10)	8	*QPRT, HSPH1, AXIN2, PMEPA1, LAPTM4B, CHMP4B, MLH1, EMP1*
Con. (CN)	Genes up-regulated after 1 h of *TGFB1* stimulation in MEF cells (embryonic fibroblast) with *NFIC* knockout vs wild type MEFs. (11)	33	*KCNAB1, SOAT1, CYP51A1, IDI1, MFAP4, FAM46C, IL2RG, DSE, INSIG1, PDGFRA, TATDN2, OXCT1, MEOX1, FOXG1, RBP1, AHR, CALCRL, NOV, DFNA5, KCNN4, RPL39L, ZFHX3, ATF4, EVI2A, EPHB2, CCR5, FLT1, ENPEP, PDP1, NR4A2, CA6, RAB39B, FLI1*
Con. (CN, all)	Genes up-regulated by RUNX1-RUNX1T1 fusion protein in normal hematopoietic progenitors; their expression was sustained in subsequently developing granulocytes. (12)	15	*GUCY1A3, SOX4, TMEM176B, ARID5B, JUP, F2RL1, ID1, CYP1A1, IL17RB, ALDH1A1, IFI16, TM4SF1, CD200, HSPB1, CRHBP*
Con. (CN, all)	Genes down-regulated in quiescent (G0) CD34+ cells isolated from peripheral blood of CML (chronic myeloid leukemia) patients compared to the quiescent cells from normal donors. (3)	47	*PCDH9, RBPMS, VNN1, HLA-DPA1, HLA-DQB1, GUCY1A3, SPINK2, FHL1, GLIPR1, SORL1, H1F0, HLA-DPB1, PPFIBP1, LIMCH1, GBP2, GPR126, HLA-DRB4, PMCH, TM4SF1, PROM1, HLF, HLX, MLLT3, TSPAN6, CXCL1, SCHIP1, CXCL6, SELL, HLA-DQA1, HIST2H2BE, AIF1, EMP1, MPZL2, KIAA0125, APP, IDS, HLA-DMA, CD52, CRHBP, AREG, TGFB1I1, HLA-DMB, DUSP6, SPTBN1, ALOX5, TFPI, BAALC*
Con. (all)	Genes down-regulated in umbilical vein endothelium cells by fenretinide.(13)	5	*CD34, PPP1R3C, LDLR, HSPA2, TFF3*

Con. (all)	Genes up-regulated in CD133+ cells (hematopoietic stem cells) compared to the CD133- cells. (14)	316	*DNMT3B, IGFBP7, GPR125, PON2, PDZD2, SCN3A, TNFRSF21, SSBP2, H2AFY, HSPD1, FRMD6, COL5A1, PSMG1, C11orf54, SCRN1, ALG10B, ZNF117, LAPTM4B, PLAGL1, ADAM28, ABCC1, ANGPT1, JUP, TRIM73, CBX2, ZNF711, HLF, PTPLA, KIAA0368, SMAD1, PRDM16, PXDN, PHACTR1, ISYNA1, FAM175A, CALN1, RAVER2, PLCB4, FRMD4B, HHEX, SLC39A10, UHRF1, TUSC1, MZB1, DST, C10orf58, DDAH1, KIAA1211, SLITRK4, WDR91, ZNFX1-AS1, SMARCA1, SLC39A8, CHST13, TAF1D, CDCA7, PAICS, ITPRIPL2, ZNRF1, KIAA0125, TRO, HDGFRP3, BTBD3, WASF1, BCAT1, DPPA4, ATP9A, TSPYL5, MSRB3, LOC400464, PDE1A, RDX, CYYR1, SOCS2, TMEM38B, RHOBTB1, BEX2, FHL1, UNG, MPL, C5orf35, ARMCX2, RCN1, KDELC1, CRISPLD1, ARHGAP22, EMP1, CRIM1, MEIS1, ARMCX1, C9orf93, EFHA2, LOC100287017, TMEM44, MYCT1, CD34, ERG, AKR1A1, ETV6, SCD, ATP6V0A2, FLVCR1, ACACA, SH3RF1, PLCB1, ZNF521, ZC3HAV1L, TXNRD3, TMEM163, ATP2C1, NME1, CCNB1IP1, EBPL, PHF16, PKD2, PM20D2, ASPH, HOXA9, MSI2, PRDX4, FLT3, TUG1, CMAHP, PRMT5, NRIP1, RBPMS, WDR49, DKC1, C11orf95, HOXB3, NAP1L3, PDGFC, UMODL1, UBTD2, HOXA3, MMP28, DTL, GOLIM4, SH3BP4, SLC25A27, CPT1A, GNA15, NPR3, CDK6, MAP7, TMEM200A, C1QTNF4, HMGCR, IL18, OBSL1, BAALC, ERLIN2, MYB, CPA3, CRHBP, TNS3, GUCY1A3, SEPP1, SRSF3, DPY19L3, TFPI, UBR5, HSH2D, NKAIN2, PLEKHA5, TFEC, HPGDS, NASP, FAM171B, DSG2, PAM, HADH, TBC1D24, IQGAP2, GALNT7, SRD5A3, ACSF2, FAM98B, KIT, ANKRD28, CTHRC1, DPY19L2, ERMP1, ZBTB8A, QSER1, ZNF709, GNAI1, RUNX2, VWDE, SPIN4, RAB34, C19orf77, HSPB1, BCL11A, FAM69B, SAMD13, ITGA9, ZBED3, SLC16A14, RUVBL2, LPIN1, CBX5, FABP5, ANKRD6, PROM1, PLS3, CDK4, ZNF302, LIMCH1, SMARCA2, FAM92A1, IPO7, CHRDL1, DPYSL3, NDN, SLC27A2, AREGB, ALDH1A1, ZNF618, SPG20, HMGA2, CD109, BIVM, RPS15A, RBM10, CREBZF, CNKSR3, AREG, C5orf13, CYTL1, PPM1H, MRPS27, SPOCK3, ZNF512B, MIR155HG, MEST, PLA2G12A, MEGF6, HOXA10, CDK2AP1, MAP9, B4GALT6, TGFBRAP1, DPY19L4, FAIM, MUM1, ADAT2, WDR17, C3orf64, DOCK7, DAPK1, MYO5C, SCHIP1, MEG3, IGLL1, C12orf24, SV2A, KCTD3, ZMAT1, F2R, ZNF165, SMYD3, CCDC6, PAIP1, DEPTOR, AKT3, SYPL1, POGZ, FAM115A, ITM2C, TCEAL4, FGD5, PSMB5, BEND4, KCTD15, NEGR1, STMN1, GATA2, NT5DC2, PLA2G4A, VAV3, MLLT3, WBP5, PGBD1, HOXA5, TRIM24, TANC1, FANCL, SOCS6, SERPING1, SPINK2, ME3, LRCH2, COL24A1, LOC100506844, TMEM5, F2RL1, TRIP6, NKX2-3, SCN9A, CEP170, MCM5, CPSF3, GPR126, BSPRY, STK3, KDM5B, IPO11, GATM, KHDRBS3, MBLAC2, MAST4, AKR1C3, GCSH, SHANK3, PREX2*

***:** IDs correspond to the node labels in **Fig. 3B**. Con: The control functional gene-set that show significant correlation with any identified gene-set only in normal HSCs (circled nodes in **Fig 3**) and are significantly associated with adverse outcomes in the bulk AML training cohort (log-rank p<0.01, coefficient >0). CN: Control gene-sets were identified by training cytogenetic normal patients. all: Control gene-sets were identified by training all AML patients.

References for Supplementary Tables 2-3:

1. Gentles, A.J., Plevritis, S.K., Majeti, R. and Alizadeh, A.A. (2010) Association of a leukemic stem cell gene expression signature with clinical outcomes in acute myeloid leukemia. *JAMA*, **304**, 2706-2715.
2. Ohashi, S., Sakashita, G., Ban, R., Nagasawa, M., Matsuzaki, H., Murata, Y., Taniguchi, H., Shima, H., Furukawa, K. and Urano, T. (2006) Phospho-regulation of human protein kinase Aurora-A: analysis using anti-phospho-Thr288 monoclonal antibodies. *Oncogene*, **25**, 7691-7702.
3. Graham, S.M., Vass, J.K., Holyoake, T.L. and Graham, G.J. (2007) Transcriptional analysis of quiescent and proliferating CD34+ human hemopoietic cells from normal and chronic myeloid leukemia sources. *Stem Cells*, **25**, 3111-3120.
4. Georgantas, R.W., 3rd, Tanadve, V., Malehorn, M., Heimfeld, S., Chen, C., Carr, L., Martinez-Murillo, F., Riggins, G., Kowalski, J. and Civin, C.I. (2004) Microarray and serial analysis of gene expression analyses identify known and novel transcripts overexpressed in hematopoietic stem cells. *Cancer Res*, **64**, 4434-4441.
5. Takeda, A., Goolsby, C. and Yaseen, N.R. (2006) NUP98-HOXA9 induces long-term proliferation and blocks differentiation of primary human CD34+ hematopoietic cells. *Cancer Res*, **66**, 6628-6637.
6. Myllykangas, S., Himberg, J., Bohling, T., Nagy, B., Hollmen, J. and Knuutila, S. (2006) DNA copy number amplification profiling of human neoplasms. *Oncogene*, **25**, 7324-7332.
7. Wang, G., Jones, S.J., Marra, M.A. and Sadar, M.D. (2006) Identification of genes targeted by the androgen and PKA signaling pathways in prostate cancer cells. *Oncogene*, **25**, 7311-7323.
8. Gavin, M.A., Rasmussen, J.P., Fontenot, J.D., Vasta, V., Manganiello, V.C., Beavo, J.A. and Rudensky, A.Y. (2007) Foxp3-dependent programme of regulatory T-cell differentiation. *Nature*, **445**, 771-775.
9. Bystrykh, L., Weersing, E., Dontje, B., Sutton, S., Pletcher, M.T., Wiltshire, T., Su, A.I., Vellenga, E., Wang, J., Manly, K.F. *et al.* (2005) Uncovering regulatory pathways that affect hematopoietic stem cell function using 'genetical genomics'. *Nat Genet*, **37**, 225-232.
10. Koinuma, K., Yamashita, Y., Liu, W., Hatanaka, H., Kurashina, K., Wada, T., Takada, S., Kaneda, R., Choi, Y.L., Fujiwara, S.I. *et al.* (2006) Epigenetic silencing of AXIN2 in colorectal carcinoma with microsatellite instability. *Oncogene*, **25**, 139-146.
11. Plasari, G., Calabrese, A., Dusserre, Y., Gronostajski, R.M., McNair, A., Michalik, L. and Mermod, N. (2009) Nuclear factor I-C links platelet-derived growth factor and transforming growth factor beta1 signaling to skin wound healing progression. *Molecular and cellular biology*, **29**, 6006-6017.
12. Tonks, A., Pearn, L., Musson, M., Gilkes, A., Mills, K.I., Burnett, A.K. and Darley, R.L. (2007) Transcriptional dysregulation mediated by RUNX1-RUNX1T1 in normal human progenitor cells and in acute myeloid leukaemia. *Leukemia : official journal of the Leukemia Society of America, Leukemia Research Fund, U.K*, **21**, 2495-2505.
13. Ferrari, N., Pfeffer, U., Dell'Eva, R., Ambrosini, C., Noonan, D.M. and Albini, A. (2005) The transforming growth factor-beta family members bone morphogenetic protein-2 and macrophage inhibitory cytokine-1 as mediators of the antiangiogenic activity of N-(4-hydroxyphenyl)retinamide. *Clin Cancer Res*, **11**, 4610-4619.
14. Jaatinen, T., Hemmoranta, H., Hautaniemi, S., Niemi, J., Nicorici, D., Laine, J., Yli-Harja, O. and Partanen, J. (2006) Global gene expression profile of human cord blood-derived CD133+ cells. *Stem Cells*, **24**, 631-641.

Table S4. Three independent primary AML studies.

**Bulk AML dataset**	**GSE14468**	**TCGA**	**GSE12417**
Journal	Blood	N Engl J Med	Blood
Year	2011	2013	2008
PMID	21937695	23634996	18716133
Last author	v der Reijden	Cancer Genome Atlas Research Network	Buske
First author	Noordermeer		Metzeler
platform	Hgu133+2	Hgu133+2	Hgu133a & Hgu133a+2
Median overall survival months in all types of AML	18.68	16.4	10.22
Median age of AML with outcome (years)	46	57	60
# of samples with outcome	518	197	242
Age (years, older than 60)	72	89	123
Sex (MALE)	261	106	NA
flt3itd	NA	64	0
del 3q	20	NA	0
del 5q	NA	15	0
del 7q	38	20	0
complex	36	24	0
inv16	42	9	0
t(8;21)	38	7	0
t(15;17)	25	16	0
Cepba	38	NA	0
t(9;11)	NA	2	0
Cytogenetically normal (CN)	214^	91	242
Median overall survival months in CN AML	19.27	16.3	10.22
Median age of CN AML with outcome (years)	48	57	60

^ excluding one "t(15:17), CN" sample

CN: cytogenetically normal

Table S5. Univariate and multivariate analyses of overall survival in patients with all types of AML, for the LSC+ DNM gene-sets.

Dataset	variate model	variates	HR	95% CI	p-value
GSE14468 (n=518)	Univariate model	**4 DNM fGSs vs 4 control fGSs**	**0.6**	**0.51-0.8**	**5.4E-5 *** **
		ELN_RiskFavorable vs. Adverse	0.3	0.20-0.40	2.15E-13 ***
		complex vs. others	2.2	1.50-3.16	0.000024 ***
		7q vs. others	2.1	1.45-2.97	0.000051 ***
		ELN_RiskIntermediate-II vs. Adverse	0.5	0.36-0.70	0.000053 ***
		Age group, years (≥60 vs. <60)	1.7	1.29-2.28	0.00016 ***
		t(8;21) vs. others	0.4	0.23-0.68	0.00046 ***
		3q vs. others	2.1	1.32-3.42	0.0015 **
		ELN_RiskIntermediate-I vs. Adverse	0.6	0.46-0.86	0.0035 **
		inv16 vs. others	0.5	0.32-0.83	0.0057 **
		cebpa mutation vs. others	0.6	0.67-0.95	0.028 *
	Multivariate model	**4 DNM fGSs vs 4 control fGSs**	**0.6**	**0.48-0.78**	**0.000063 *** **
		Age group, years (≥60 vs. <60)	1.7	1.22-2.22	0.00097 ***
		ELN_RiskFavorable vs. Adverse	0.4	0.24-0.74	0.0026 **
		complex vs. others	1.4	0.86-2.38	0.17
		inv16 vs. others	0.7	0.40-1.21	0.20
		ELN_RiskIntermediate-II vs. Adverse	0.7	0.42-1.23	0.22
		cebpa mutation vs. others	0.8	0.49-1.34	0.41
		3q vs. others	1.3	0.70-2.24	0.44
		7q vs. others	1.1	0.70-1.88	0.59
		ELN_RiskIntermediate-I vs. Adverse	0.9	0.51-1.48	0.60
TCGA (n=197)	Univariate model	**4 DNM fGSs vs 4 control fGSs**	**0.73**	**0.52-1.00**	**0.051 .**
		Age group, years (≥60 vs. <60)	3.02	2.16-4.21	9.94E-12 ***
		gender	0.88	0.64-1.22	0.44
		normal_karyotype vs. others	1.12	0.81-1.55	0.50
		BM Blast(>50 vs. <=50)	0.88	0.60-1.30	0.53
	Multivariate model	**4 DNM fGSs vs 4 control fGSs**	**0.83**	**0.60-1.16**	**0.272**
		Age group, years (≥60 vs. <60)	2.94	2.10-4.11	3.1E-10 ***
GSE12417 (n=242)	Univariate model	**4 DNM fGSs vs 4 control fGSs**	**0.49**	**0.36-0.69**	**2.48E-5 *** **
		Age group, years (≥60 vs. <60)	1.63	1.18-2.26	0.0029 **
	Multivariate model	**4 DNM fGSs vs 4 control fGSs**	**0.53**	**0.38-0.73**	**0.00016 *** **
		Age group, years (≥60 vs. <60)	1.49	1.08-2.08	0.016 *

Significance code: ' .':p<.1; '*': p<.05; '**'p<.01; '***'p<.001

Significant univariate tested factors (p<.05) are used for multivariate test, the independent significant factors are listed in black whereas the dependent factors are listed in grey.

Table S6: The somatic mutations (of genes in the identified DNM gene-sets) and their patient information in the TCGA database.

Gene member	DNM fGS cluster	# of pts with mutation	Variant_Classification	mutation corrdiante	# of pts with gene expression	TCGA Patient ID
**DDX53**	LSC-	1	Missense	chrX:22928217	1	
**ETV6**	LSC+	2	Missense/Frame_Shift_Del	chr12:11930226;11883488	2	2891,3012
**KIF1C**	LSC+	1	Nonsense	chr17:4846516	1	
**KRAS**	LSC+	8	Missense	chr12:25271542;25289551;25271549;25289551;25289478;25289551;25269829;25289548	7	2826,2861,2864,2865,2917,2966,2987
**NAV1**	LSC+	2	Silent	chr1:200043815,199884746	2	2905,3009
